# A generalized analysis of hydrophobic and loop clusters within globular protein sequences

**DOI:** 10.1186/1472-6807-7-2

**Published:** 2007-01-08

**Authors:** Richard Eudes, Khanh Le Tuan, Jean Delettré, Jean-Paul Mornon, Isabelle Callebaut

**Affiliations:** 1 Structural Biology Department, IMPMC, CNRS UMR7590, Universités Paris 6 & Paris 7, case 115, 4 place Jussieu, 75252 Paris Cedex 05, France

## Abstract

**Background:**

Hydrophobic Cluster Analysis (HCA) is an efficient way to compare highly divergent sequences through the implicit secondary structure information directly derived from hydrophobic clusters. However, its efficiency and application are currently limited by the need of user expertise. In order to help the analysis of HCA plots, we report here the structural preferences of hydrophobic cluster species, which are frequently encountered in globular domains of proteins. These species are characterized only by their hydrophobic/non-hydrophobic dichotomy. This analysis has been extended to loop-forming clusters, using an appropriate loop alphabet.

**Results:**

The structural behavior of hydrophobic cluster species, which are typical of protein globular domains, was investigated within banks of experimental structures, considered at different levels of sequence redundancy. The 294 more frequent hydrophobic cluster species were analyzed with regard to their association with the different secondary structures (frequencies of association with secondary structures and secondary structure propensities). Hydrophobic cluster species are predominantly associated with regular secondary structures, and a large part (60 %) reveals preferences for α-helices or β-strands. Moreover, the analysis of the hydrophobic cluster amino acid composition generally allows for finer prediction of the regular secondary structure associated with the considered cluster within a cluster species. We also investigated the behavior of loop forming clusters, using a "PGDNS" alphabet. These loop clusters do not overlap with hydrophobic clusters and are highly associated with coils. Finally, the structural information contained in the hydrophobic structural words, as deduced from experimental structures, was compared to the PSI-PRED predictions, revealing that β-strands and especially α-helices are generally over-predicted within the limits of typical β and α hydrophobic clusters.

**Conclusion:**

The dictionary of hydrophobic clusters described here can help the HCA user to interpret and compare the HCA plots of globular protein sequences, as well as provides an original fundamental insight into the structural bricks of protein folds. Moreover, the novel loop cluster analysis brings additional information for secondary structure prediction on the whole sequence through a generalized cluster analysis (GCA), and not only on regular secondary structures. Such information lays the foundations for developing a new and original tool for secondary structure prediction.

## Background

Prediction of secondary structures is a fundamental basis for protein structure prediction. This provides constraints for finding remote homologues with low sequence similarity for comparative modeling (*e.g*. [1]) and starting points for fold recognition (*e.g*. [2]). This information is particularly useful to infer biological function from the expanding sequence data originated from genomes, as the gap between sequences and experimental structures is continuously growing.

Current prediction methods generally extract information from known experimental structures and use it for predicting secondary structures in unknown sequences. A substantial improvement in secondary structure prediction has been made by taking into account the evolutionary information provided by the divergence of protein sequences belonging to a same structural family (*e.g*. [2,3], reviewed in [4]). Predictions now reach accuracy around 75–80 % of all residues predicted correctly on the basis of three (alpha, beta, coil) states (three-state per residue-based accuracy). Accuracy limitation may come from inconstancies between the different secondary structure assignment methods [5], but also from long-range interactions, which are not considered in current predictive tools [6,7].

Implicit information about secondary structure can be efficiently considered in the sequence comparison by using an original, lexical approach, called Hydrophobic Cluster Analysis (HCA) [8,9]. This information can be directly unraveled from the analysis of the primary structure and without necessarily use of multiple alignments. Hydrophobic clusters delineated using HCA are indeed statistically centered on the regular secondary structure elements, whatever their nature maybe (alpha-helix or beta-strand) [10]. The definition of hydrophobic clusters delineated through HCA relies on two parameters: the **hydrophobic alphabet **and the **connectivity distance**, which sets up the minimal number of non-hydrophobic amino acids separating two different clusters. This HCA connectivity distance originates from the constant curvature of the 1D sequence space into the Euclidian three-dimensional space along a helical path, and from the associated use of a two-dimensional support to represent the protein sequence. The VILFMYW alphabet and the connectivity distance of 4 (corresponding to the α-helix curvature) allow the better correspondence between the hydrophobic clusters and regular α or β secondary structures [10]. The VILFMYW alphabet is also supported by the greater propensities of these residues to be included in regular secondary structures than in coils [9], as well as by their general burying [11-13]. An interesting feature of hydrophobic clusters, due to the use of a connectivity distance constraint, is that they cannot be intertwined, *i.e*. they cannot include or be included in any other hydrophobic clusters. As a consequence, hydrophobic clusters are considerably better markers of regular secondary structures than simple binary patterns of hydrophobic/non hydrophobic residues, which do not depend on a connectivity distance [14].

The power of HCA in revealing the position and often the nature of regular secondary structures from the analysis of a single amino acid sequence makes it an efficient tool for comparing sequences of distantly related proteins, identifying remote relationships and deciphering orphan sequences (*e.g*. [15-19]; see [20] for a list of investigations performed by our group). The secondary structure compatibility of the compared sequences can be rapidly estimated, and importantly, the limitations of alignments provided by standard similarity search programs, especially for the handling of indels, can often be overcome. Indeed, HCA does not suffer from the presence of indels, even if they are large (*e.g*. domain insertion). Of note is the accuracy of secondary structure information that can generally be obtained about orphan sequences, for which no homologue can be identified in databases by standard similarity searches, or sequences having only close homologues.

However, the efficiency of HCA largely depends on the user expertise, which has also hampered heretofore its application for large-scale genome analyses.

The general correspondence between hydrophobic clusters, taken as a whole, and regular secondary structures has been demonstrated several years ago [10], but no detailed analysis of their individual structural behaviors and preferences for α or β secondary structures has yet been reported. Here, we describe the frequencies of association with secondary structures and secondary structure propensities of 294 hydrophobic cluster species, defined only by their dichotomy in hydrophobic/non-hydrophobic residues, and which are frequently observed in protein globular domains. The resulting dictionary can help to interpret the HCA plots of protein sequences and to compare them. The observed secondary structures of hydrophobic cluster species typically associated with α helices and β strands were also compared with the predictions made on the basis of current tools, such as PSI-PRED [21]. Finally, we also investigated the behavior of loop forming clusters, using an appropriate loop alphabet with the same connectivity distance as for hydrophobic clusters. Such investigation may bring additional information for secondary structure prediction on the whole sequence using a generalized cluster analysis (GCA), and not only on regular secondary structures. They also lay the foundations for developing a HCA-based, automatic tool for secondary structure prediction.

## Results

### Hydrophobic cluster analysis

#### Definition of hydrophobic clusters

The principles of Hydrophobic Cluster Analysis (HCA) have been previously detailed [8,9]. Briefly, HCA relies on a helical curvature of the "1D" representation of the amino acid sequence in a space of higher dimensionality (3D). This allows the detection and visualization, through a 2D transposition of the sequence (the HCA plot), of the local hydrophobic compactness (hydrophobic clusters) largely associated with internal faces of regular secondary structures (α-helices and β-strands). Hydrophobic clusters definition depends on three parameters: i) a representative hydrophobic alphabet, ii) an optimal helical pitch to curve the 1D amino acid sequence space in a constant way, iii) a connectivity distance, depending on the considered helical pitch and corresponding to the number of non-hydrophobic residues separating two hydrophobic clusters defined as distinct. Seven hydrophobic residues (V, I, L, F, M, Y, W) integrate the HCA hydrophobic alphabet. These residues are, with cysteine, the most buried [11-13] and are more often associated with regular secondary structures (RSS, α-helices and β-strands) than with coils [9]. The optimal helical pitch (for both α and β RSS), assessed by the best correspondence between RSS and hydrophobic clusters [10], is the α-helix pitch, with an associated connectivity distance of 4 amino acids (approximately one helix turn).

**Figure 1:**
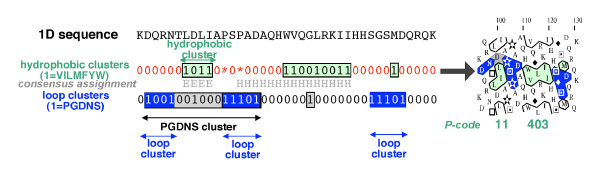
**Definition of hydrophobic and loop clusters**. The sequence of the C-terminal end of the phospholipase C δ PH domain, whose structure has been experimentally solved (pdb 1mai), is shown as example. The sequence is translated into two binary sequences, using on the one hand, a hydrophobic cluster specific-code (1 = VILFMYW, * = P, 0 = other residues) and on the other hand, a loop cluster specific-code (1 = PGDNS, 0 = other residues). The consensus assignment of secondary structures is shown between the two binary sequences (E = strand, H = helix). On the binary-encoded sequences, hydrophobic clusters are shaded in green (1 = VILFMYW), whereas PGDNS and loop clusters are colored in grey and blue, respectively (1 = PGDNS). Loop clusters are necessarily included in PGDNS clusters (the blue color superimposes with the grey one), as information brought by the hydrophobic clusters has been omitted from the PGDNS clusters to form loop clusters. Loop clusters and hydrophobic clusters are therefore non-intertwined. Hydrophobic clusters and loop clusters always begin and end with "1". The positions encoded by 1 within clusters (hydrophobic, PGDNS and loop clusters) are colored accordingly on the 2D HCA representation, shown at right. The Peitsch codes (P-codes) of the two hydrophobic clusters are indicated at bottom. Note that the α-helix is longer than the corresponding hydrophobic cluster (6 amino acids upstream). However, this sequence includes two alanine residues, which have strong propensities for helical structure (*e.g*. [9]). Alanine has not been integrated into the hydrophobic alphabet, as it does not increase the global mean correspondence between hydrophobic clusters and regular secondary structures (data not shown). This amino acid has to be considered in a context-dependant way. HCA plots were drawn using DrawHCA [43].

Strong hydrophobic amino acids (V, I, L, F, M, Y, W) are coded 1, while others are coded 0 (Figure 1). Then, any hydrophobic cluster begins and ends with 1 and no stretch of 0, of length superior or equal to the connectivity distance, can be found within a cluster. Consequently, two distinct hydrophobic clusters are separated by at least four 0 (standard connectivity distance) or any sequence segment containing a proline. Indeed, this particular residue appears to be in itself a strong RSS breaker. Underlined fonts (*e.g*. ***10101***) are used to distinguish clusters from ordinary binary patterns, which may be embedded in larger patterns (*e.g*. 10101 in 110101) (see [14] for a comparison of hydrophobic clusters and standard binary patterns). Following these simple rules, all combinations of 0 and 1 give rise to different hydrophobic cluster species. Each species may thus gather very different sequences, which are however all characterized by the same hydrophobic/non-hydrophobic binary pattern. To depict a particular cluster species, two convenient codes are used in addition to the binary code: the decimal translation of the binary code, also named "Peitsch code" or "P-code" (defined as the sum of the powers of 2, indexed according to the position of each number of the binary code (the last position corresponds to 0), each power being multiplied by the binary code value; *e.g*. ***110101***=> 1 × 2^5 ^+ 1 × 2^4 ^+ 0 × 2^3 ^+ 1 × 2^2 ^+ 0 × 0^1 ^+ 1 × 2^0 ^= 53) and the Q-code, which considers clusters as concatenations of the four following basic clusters: ***11***V (for Vertical), ***101***M (for Mosaic), ***1001***U (for Up) and ***10001***D (for Down) (*e.g*. ***10101***= ***101***+ ***101***= MM). The Q-code was not used in this report, in contrast to the P-code. The P-code indeed allows an easy alternative description of clusters, which is useful in terms of computational storage and classification procedures. In this context, and in particular for long clusters, the P-code is more convenient to memorize and manipulate than the binary code. A binary/P-code converter is available at [22]. The number of hydrophobic cluster species exponentially increases with the cluster length, while the observed occurrences of hydrophobic clusters within species concurrently decrease.

#### Redundancy treatment

Redundancy of databases must be reduced to avoid statistical bias. At the same time, working with weakly redundant databases does not allow valuable statistics on a large number of cluster species. For example, just 97 hydrophobic cluster species are represented at least 30 times in our 5 % database (*in which sequences do not share more than 5 % identity with any other sequence of the bank*), against 150, 250 and 304 in the 25 %, 50 % and 90 % databases, respectively (see Additional file 1). It is however interesting to note that even at very low level of redundancy (5%), the 97 informative hydrophobic cluster species gather a large fraction of the total number of hydrophobic clusters in the bank (73 %, against 76 % and 81 % in the 25 % and 90 % databases, respectively). If the simplest and highly populated hydrophobic cluster species *1*(P-code 1) and *11*(P-code 3) are omitted from this calculation (*these are indeed weakly associated with regular secondary structures – see below*), the remaining hydrophobic clusters belonging to informative species (with at least 30 members) totalize 61%, 65 % and 73 % of the total numbers of hydrophobic clusters of the 5 %, 25 % and 90 % databases, respectively. However, the use of banks with higher redundancy extends the set of informative cluster species to higher lengths. Hence, whereas only 15 informative cluster species of length 9 can be exploited at 5 %, 70 and 64 cluster species of length 9 and 10, respectively, are available at 90 % redundancy, and a few cluster species can be found up to length 15 (see Additional file 1). This allows getting statistics for clusters associated not only with β-strands, but also with many α-helices.

A practical way to solve the redundancy problem with regard to cluster species is to select, species by species, the appropriate level of redundancy by observing the evolution of the hydrophobic cluster occurrence within a given species as a function of redundancy. Abrupt thrust in the curves, translating the existence of very similar sequences, can easily be visualized (see Additional file 2, illustrating a large set of clusters (*top panel*), for which occurrences are normalized relative to the values observed in the 50 % database, taken as a reference, as well as the particular case of two clusters (*bottom panel*). *The 50 % level was chosen as a reference because it roughly corresponds to the inflection point of the curves reporting, species by species, cluster occurrences as a function of the redundancy level. Moreover, this mid value allows the similar handling of extreme levels of redundancy (5 % and 95 %)*). For example, occurrences of hydrophobic clusters corresponding to the P-code 153 (*10011001*), which are predominantly associated with α-helical structures (see below), grow continuously as a function of redundancy, whereas occurrences of hydrophobic clusters of species 137 (*10001001*, also predominantly associated with alpha-helices), suddenly grow faster from 80% of redundancy (see Additional file 2, *bottom panel*). As a consequence, we selected the 90% redundancy statistics for cluster species behaving like 153 (88 % of the total number of species), whereas we chose the appropriate lower level of redundancy for others (no less than 70 %, *e.g*. 75 % was chosen for the species 137). Ten cluster species out of the 304 that were initially considered in the 90 % database were discarded, to reach a final number of 294. The chosen levels are indicated in the table reporting the structural preferences of these 294 hydrophobic clusters, supplied as Additional file 3 (also see [23]).

**Figure 2:**
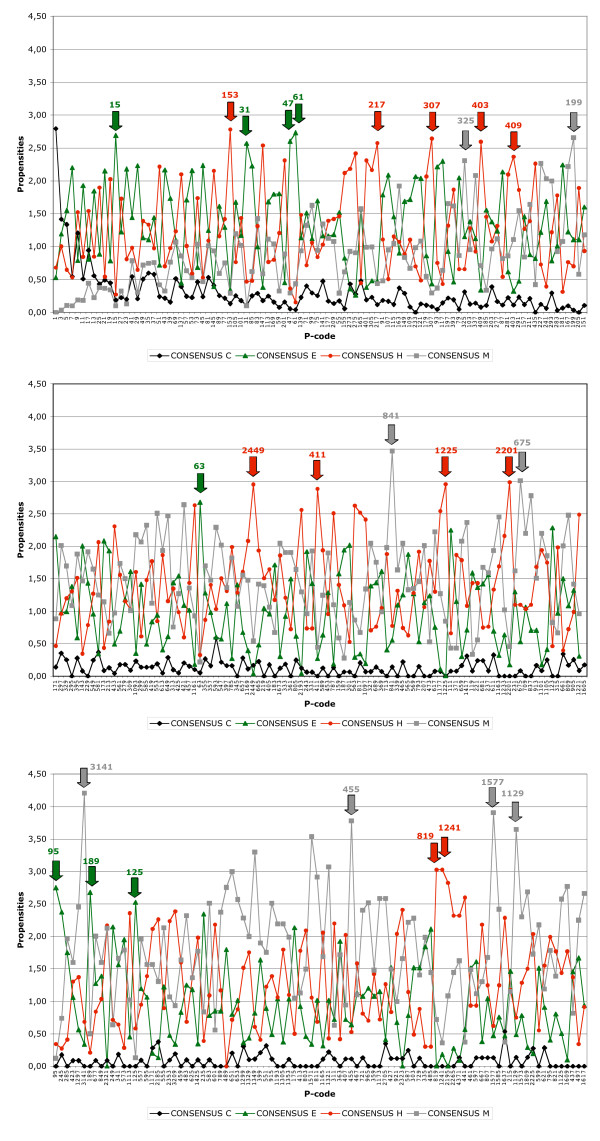
**Hydrophobic cluster propensities for the three secondary structures (α,β and coil) and for «multiple» clusters (clusters associated with at least two different regular secondary structures)**. Hydrophobic cluster species are classified in the decreasing order of their occurrences from the first to the third panels. Propensities are only reported for the chosen High Redundancy (HR) database (90 %, at the exception of a few hydrophobic clusters for which lower levels of redundancy were chosen – Additional file 3). Similar propensities were observed in the 25% database, when the occurrence of clusters within the considered species is yet sufficient to allow accurate statistics. Several cluster species are highlighted with arrows, with respect to their noticeable propensities for strand, helix or coil structures.

**Figure 3:**
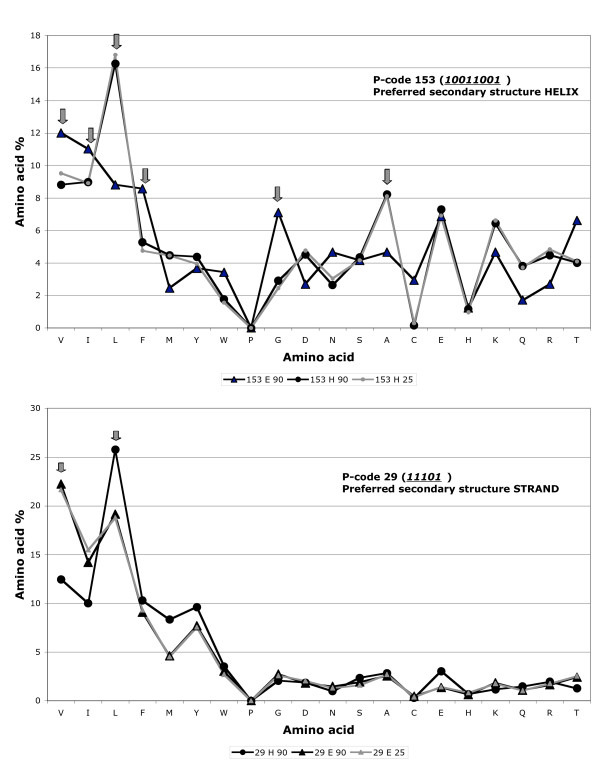
**Amino acid composition of the two (preferred and non-preferred) regular secondary structures associated with two typical hydrophobic cluster species**. The preferred secondary structures of hydrophobic cluster species 153 and 29 correspond to the alpha-helix and to the beta strand, respectively. Values are indicated for the preferred and non-preferred secondary structures within the 90% redundancy database (black), and only for the preferred secondary structure within the 25 % database (grey). Within the 25 % database, the cluster occurrence is indeed too low for the non-preferred secondary structure. **153**: 90% level: 461 clusters (81.1 % helices and 11.1 % strands); 25% level: 211 clusters (79.6 % helices and 9.5 % strands). **29**: 90% level: 1079 clusters (18.9 % helices and 71.4 % strands); 25% level: 498 clusters (18.5 % helices and 72.9 % strands). Arrows indicate noticeable behaviors of amino acids, with respect to helices or strands.

The observed features of hydrophobic cluster species are remarkably stable relative to redundancy, as exemplified by constant frequencies of associations with secondary structures (see Additional file 4, example of two hydrophobic cluster species at all levels of redundancy), by similar secondary structure propensities (Figure 2) and amino acid compositions (Figure 3, example of two hydrophobic cluster species at the 25 % and high redundancy levels). Thus, these observations subsequently support the choice of high redundancy databases.

#### Association of hydrophobic clusters with secondary structures

Each hydrophobic cluster species was analyzed with regard to its association with secondary structures. Three different algorithms (DSSP, STRIDE, PSEA) were used to assign secondary structures (helix (H), strand (E), coil (C)) from atomic coordinates. Assignments given in the PDB files (SSPDB) were also considered. A consensus assignment was deduced from the four assignment methods and the results obtained on the basis of the different assignment methods were similar. Furthermore, with respect to the different assignments methods, good correlation coefficients were indeed observed when considering, as variables, the frequencies of association with secondary structures (H, E, C) of the different cluster species (Table 1). Good correlation coefficients were also obtained for secondary structure propensities (Table 2). We detail below results obtained with the consensus assignment.

**Table 1:** Frequencies of association of hydrophobic clusters with secondary structures: comparison of the different methods for secondary structure assignment.

**α/β/C**	**Consensus**	**SSPDB**	**DSSP**	**STRIDE**	**PSEA**
**Consensus**	1/1/1	0.99/0.99/0.98	0.99/0.99/0.99	0.99/0.99/0.92	0.99/0.99/0.99
**SSPDB**		1/1/1	0.99/0.99/0.98	0.99/0.98/0.91	0.99/0.99/0.99
**DSSP**			1/1/1	0.99/0.98/0.90	0.99/0.99/0.99
**STRIDE**				1/1/1	0.99/0.98/0.91
**PSEA**					1/1/1

Correlation coefficients were calculated with respect to the frequencies of association of the 294 hydrophobic cluster species reported in Additional file 3 with secondary structures (H, E, C; APC rule), following secondary structure assignment from different methods (SSPDB, DSSP, STRIDE, PSEA, consensus). The three values reported in the cells of this table correspond to the correlation coefficients calculated with respect to the different secondary structures (alpha, beta, coil).

**Table 2:** Propensities of hydrophobic clusters for secondary structures: comparison of the different methods for secondary structure assignment.

**α/β/C/M**	**SSPDB**	**STRIDE**	**DSSP**	**PSEA**	**Consensus**
**SSPDB**	1/1/1/1	0.99/0.93/0.97/0.83	0.99/0.94/0.98/0.86	0.97/0.92/0.79/0.77	0.99/0.94/0.98/0.86
**STRIDE**		1/1/1/1	0.99/0.99/0.98/0.97	0.97/0.95/0.80/0.79	0.99/0.99/0.98/0.97
**DSSP**			1/1/1/1	0.97/0.94/0.79/0.78	0.99/0.99/0.99/0.99
**PSEA**				1/1/1/1	0.97/0.95/0.79/0.79
**Consensus**					1/1/1/1

Correlation coefficients were calculated with respect to the secondary structure propensities of the 294 hydrophobic cluster species reported in Additional file 3 with secondary structures (H, E, C, M; OPS rule), following secondary structure assignment with different methods (SSPDB, DSSP, STRIDE, PSEA, consensus). The four values reported in the cells of this table correspond to the correlation coefficients calculated with respect to the different secondary structures (alpha, beta, coil, multiple).

Two different ways were considered to analyze the association of a hydrophobic cluster species with secondary structures (*see Material and Methods*). On one hand, a "raw mean" can be deduced by calculating the strict percentages of H, E and C assignments within the cluster limits (the APC rule for "all positions considered"). This rule has the advantage of accounting for all cluster positions and, in particular, of revealing "strong" cluster species for which the majority of positions are associated with a defined regular secondary structure (*e.g*. in Additional file 3: species 15 (*1111*) and 31 (*11111*), for which the frequencies of association with β-strands according to the APC rule are 81 and 78, respectively). The APC rule has however two disadvantages: i) the signal associated with regular secondary structures tends to be "faded" by the coil signal coming from the cluster borders, as the limits of hydrophobic clusters often do not exactly correspond to those of regular secondary structures; ii) it is impossible to estimate whether there is *on average *a single regular secondary structure associated to the hydrophobic cluster or several ones. Another way to proceed and overcome these limitations is to consider that if within a cluster one or more contiguous amino acids are assigned H (or E), the entire cluster is assumed to be associated with a helix (or with a strand) (the OPS rule for "one position is sufficient"). This excludes the very few clusters containing amino acids that are associated with the two regular secondary structures (helix and strand), as well as the more numerous clusters that contain at least two different strings of regular secondary structures separated by coil positions. These peculiar clusters are called multiple (M) and are considered separately. The main artifact that may arise from this OPS rule is a potential poor coverage of the hydrophobic cluster limits by the regular secondary structures. We therefore calculate, for each species, the average rates of residues assigned H (α-coverage) or E (β-coverage) within the hydrophobic cluster limits, as described in the Methods section. This coverage is close to a Q_i _value, calculated on the cluster limits, but differ from SOV values, which would take into account the overflowing of the observed regular secondary structures outside the cluster limits. We frequently observed high coverage values (>70 %, Additional file 3), in particular for helices (93.2 % of the observations reported in Additional file 3, versus 45.7 % for the strands (this calculation excludes species *1*, for which the coverage value is obviously 100 % and species for which the considered regular secondary structure is not observed)). The resulting mean values are high (83.5 % (α) and 68.2 % (β)), revealing a good coverage of the hydrophobic clusters by regular secondary structures. It is worth noting that coverage values are, on average, lower for strands. For lengths lower or equal to the mean length of β-strands (6 residues), there is however no difference, on average, between α-helix and β-strand coverage values associated with the same hydrophobic cluster species. Differences appear for higher lengths: the strand assignments do not cover, on average, all the hydrophobic cluster positions. Relatively low values can also originate from the shift frequently observed between the gravity centers of regular secondary structures and hydrophobic clusters (0.6 and 0.3 residues on average towards the N-terminus for α-helices and β-strands, respectively (mean standard deviations σ_α _= 2.8 and σ_β _= 2.1, respectively; association with regular secondary structures assigned using the OPS rule)). This shift can exacerbate differences observed between α-helix and β-strand coverage values, mainly resulting from their different mean lengths. Finally, the correlation coefficients calculated between APC and OPS percentages reported in Additional file 3 indicate that these variables are highly correlated (0.96 (helices) and 0.93 (strands)).

**Figure 4:**
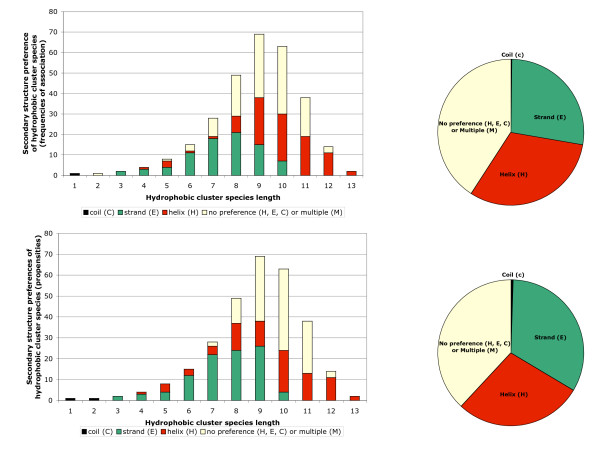
**Structural preferences of the 294 hydrophobic cluster species**. Distribution of the hydrophobic cluster species as a function of cluster length (*left*) and global distribution of hydrophobic species (*right*). The different colors indicate the structural preferences of hydrophobic cluster species and were deduced: i) from the frequencies of association with secondary structures, using the OPS rule (maximal values, shaded green in Additional file 3), ii) from the secondary structure propensities (affinity column in Additional file 3).

The lengths of hydrophobic cluster species reported in Additional file 3 vary from 1 to 13 (Figure 4). However, all the possible hydrophobic cluster species are similarly not represented above length 6. For example, species 127 (*1111111*) is not present in Additional file 3, because of a too low occurrence (5 in the 90 % database). Such hydrophobic clusters, which are not optimally balanced in hydrophobic/non-hydrophobic residues or which are too long are indeed rare. The number of sufficiently populated cluster species (for current statistics) is maximal for length 9 (69 hydrophobic cluster species), whereas above this length, the number of species reduces progressively, to reach 2 at length 13.

For many hydrophobic clusters, there is a clear preference for a particular regular secondary structure (called preferred secondary structure of a cluster species). This is illustrated in Additional file 3, which reports the percentages of association of each hydrophobic cluster species with secondary structures (using the APC and OPS rules described above) and secondary structure propensities, calculated using the OPS rule as described in Material and Methods. These structural preferences are detailed in Figure 4, which illustrates the numbers of hydrophobic cluster species that are preferentially associated with the different secondary structures (with respect to frequencies of association with secondary structures (*top*) and secondary structure propensities (*bottom*)). Hence, it can be observed that 60 % of the hydrophobic cluster species analyzed in this study shows preference either for helices or for strands (preferred secondary structure of the cluster species). The secondary structure propensities are also illustrated in Figure 2, in which cluster species are classified according to their occurrence in the 90 % database.

Contrasting with the vast majority of hydrophobic clusters, which are mainly associated with regular secondary structures (mainly with helices, mainly with strands or with either helices or strands), the smallest hydrophobic clusters (*1*and *11*; P-codes 1, 3), which contain too few amino acids to constitute stable elements of regular secondary structures, are mainly associated with coils (Additional file 3). Also notable is the relative low level of association with regular secondary structure of the basic hydrophobic clusters V (*11*; P-code 3), M (*101*; P-code 5), U (*1001*; P-code 9) and D (*10001*; P-code 17), on the basis of which all cluster species can be built. These hydrophobic clusters are also not sufficiently rich in hydrophobic residues to constitute, by themselves, stable regular secondary structures. In the dictionary presented in Additional file 3, the total frequency of hydrophobic clusters associated with coils, following the OPS rule, is 28.1% (the small clusters, which are preferentially associated with coils, are also highly populated) whereas, when omitting for the calculation hydrophobic clusters with P-codes 1, 3, 5, 9 and 17, this frequency falls to only 6.5 %.

**Figure 5:**
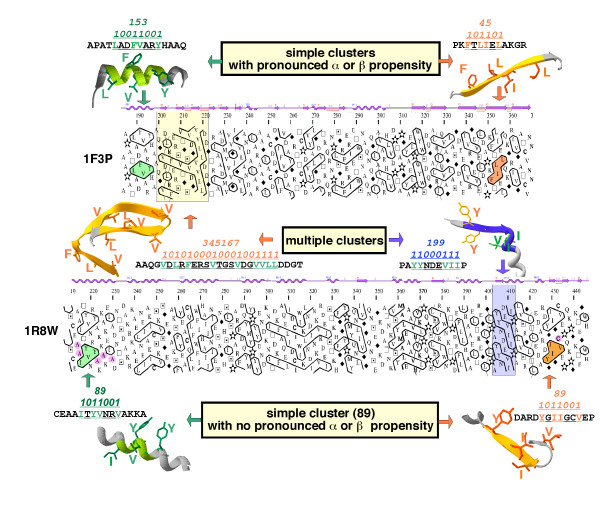
**Several examples of hydrophobic clusters**. ***Top panel***: Example of two hydrophobic clusters, found in ferredoxin reductase (pdb 1f3p) and mainly associated with helices (P-code 153) and strands (P-code 45), respectively. These hydrophobic clusters are colored on the corresponding 3D representation, in which side chains of hydrophobic residues, constituting the internal face of regular secondary structures, are shown in atomic details. Note that the alpha-helix is larger than the corresponding hydrophobic cluster, alanine residues present upstream and downstream from the cluster contributing to the extension of the alpha helix outside the cluster limits. ***Bottom panel***: Example of two hydrophobic clusters within a same protein sequence, which belong to the same species (P-code 89) but are associated with two different regular secondary structures (glycerol dehydrogenase, pdb 1r8w). The chemical nature of residues in the vicinity of hydrophobic clusters can orientate the secondary structure prediction. For instance, five alanine residues in the vicinity of the first hydrophobic cluster are indicative of a helical structure, whereas the cysteine embedded within the second hydrophobic cluster reinforces the probability of a β-strand. In addition to these examples of simple clusters, two "multiple" hydrophobic clusters are shown. These are associated with more than one regular secondary structure (P-codes 345167 and 199). The secondary structure assignments, as deduced from DSSP [31], are shown above the plots.

Outside these basic or very small clusters, preferences for α-helices or β-strands can generally be observed for many hydrophobic clusters. Maximal β-strand frequencies (OPS rule) are observed for cluster species *1111*(P-code 15: 86 %), *111101*(P-code 61: 87 %), *111111*(P-code 63: 86 %), *101111*(P-code 95: 88 %) and *10111101*(P-code 189: 86 %). Maximal α-helix frequencies are observed for cluster species *10011001*(P-code 153: 81 %), *110011011*(P-code 411: 84 %), *1100110011*(P-code 819: 88 %), *10011001001*(P-code 1225: 86 %) and *100110010001*(P-code 2449: 86 %). Note that most of these helix and strand-specific hydrophobic clusters include the basic *10011001*and *1111*hydrophobic clusters, respectively. Small and large hydrophobic clusters included in the dictionary show preference for β-strands and α-helices, respectively, whereas no preference for a particular regular secondary structure or a predominance of high affinities for the "multiple" state (*i.e. hydrophobic clusters which are associated with more than one regular secondary structure*) can generally be observed for intermediate lengths (between lengths 9 and 11, Figure 4). The secondary structure preferences are essentially related to the geometrical shapes of hydrophobic clusters on the 2D HCA transposition. As a rule of thumb, long, horizontal clusters are mainly associated with amphipatic α-helices, whereas short, vertical shapes mainly correspond to β-strand. As exemplified in Figure 5 (top panel), *10011001*(P-code 153) and *101101*(P-code 45) essentially correspond to α-helices (81 %) and β-strands (71 %) (OPS rule), respectively (propensities values of 2.8 and 2.2, respectively; α-coverage and β-coverage of 81 % and 80 %, respectively). These features can be explained by simple structural considerations. Indeed, the progression per residue along the main axis of a strand is near 3.5 Å, whilst it is only 1.5 Å for helices. As a consequence, in order to participate to a similar extent in globular domains, helices need twice more hydrophobic and/or hydrophilic residues and give rise to longer hydrophobic clusters than β-strands. As amphiphilic helices are much more frequent than internal ones, the corresponding clusters have generally horizontal shapes, owing to the periodicity of the associated binary pattern. Clusters of intermediate shape, for which no clear intrinsic periodicity of α-helices or β-strands can be evidenced, are more equi-distributed between the two regular secondary structures. For example in Figure 5 (bottom panel), *1011001*(P-code 89) is associated at 35 % with α-helices and at 50 % with β-strands (OPS rule; α and β-coverage of 89.6 and 77.7 %, respectively). This last hydrophobic cluster begins with the canonical strand pattern 101 and is followed by a canonical helix pattern 1001.

It is important to note that the 2D shapes on the HCA plot of hydrophobic clusters associated with α-helices are almost identical to their actual 3D counterparts within the protein architecture. Indeed, the standard support of HCA to curve the 1D space is the α-helix (connectivity distance (CD) 4) and thus, there is a direct correspondence between 2D and 3D hydrophobic clusters. The hydrophobic cluster with P-code 153, shown in Figure 5, illustrates this feature. For clusters associated with β-strands (*e.g*. P-code 45 in Figure 5), there is also quite good shape conservation between hydrophobic clusters on the 2D HCA plot and on the actual 3D structure. This is because the extended structure is mathematically a 2D degenerated helix with a connectivity distance of 2 (CD 2) [9]. Therefore, the HCA transposition offers at a glance the actual or nearly actual shape of the internal faces of α and β regular local structures.

The secondary structure percentages and propensities described in Additional file 3 may thus provide a direct and simple way to predict the likely secondary structure associated with a hydrophobic cluster within a single sequence. Moreover, considering the chemical nature of amino acids belonging to the hydrophobic clusters (as well as to their neighborhoods) generally allows the refinement of secondary structure prediction associated with the cluster species. Indeed, differences in the global amino acid composition can be observed between the preferred secondary structure state (helix or strand) and the other state associated with each cluster species, as exemplified in Figure 3, which illustrates two representative clusters mainly associated with alpha helices and beta-strands, respectively. Indeed, a clear preference for leucine and alanine is observed for α-helix configuration (preferred secondary structure state) of cluster species 153 (*10011001*), whereas glycine and cysteine are more frequent in the β-strand configuration. Similar trends are observed for the β-strand-associated species 29 (*11101*), for which leucine is preferred in the α-helix configuration, whereas valine and isoleucine are more frequent in the β-strand configuration (preferred secondary structure state).

"Multiple clusters", which are associated with at least two regular secondary structures, are frequently encountered within some hydrophobic cluster species. They are logically observed in long clusters (*e.g*. cluster species 1577 (*11000101001*), 64 % multiple (multiple propensity of 3.91)) but are also detected in clusters of relatively small sizes (*e.g*. cluster species 199 (*11000111*) in Figure 5 (bottom panel), or 325 (*101000101*), 43 % and 38 % multiple, respectively). The regular secondary structures covered by the cluster limits are generally separated by short loops or can even be contiguous (*e.g*. cluster 199 in Figure 5 (bottom panel)). As a rule of thumb, multiple clusters most often include a stretch of three contiguous non-hydrophobic residues (0), indicating the presence of a short loop. The loops included in larger multiple clusters generally contain an isolated hydrophobic residue, which makes the bridge between the different parts of the hydrophobic cluster matching the regular secondary structures. An example of such a scenario is observed in Figure 5 (top panel) for cluster *1010100010001001111*(P-code 345167, not included in Additional file 3 due to a too low occurrence). For clusters of sufficient length, changes in the periodicity in hydrophobic/non-hydrophobic residues are generally indicative of the coverage of several regular secondary structures. These changes (or non-homogeneity) can reveal the transition from a strand to a helix (from a periodicity in hydrophobic residues of 2 to a periodicity of 3/4) or between two strands (two high densities in hydrophobic residues separated by a region containing less hydrophobic residues (cluster 345167 *1010100010001001111*in Figure 5 (top panel)).

Finally, one can also note that symmetric hydrophobic clusters generally possess similar structural behaviors (see for example the symmetric clusters of length 6 : **35 **(*100011*; 39 % α)/**49 **(*110001*; 41 % α); **37 **(*100101*; 46 % β)/**41 **(*101001*; 46 % β); **39 **(*100111*; 55 % β)/**57 **(*111001*; 55 % β); **43 **(*101011*; 69 % β)/**53 **(*110101*; 69 % β); **55 **(*110111*; 49 % β)/**59 **(*111011*; 58 % β); **47 **(*101111*; 83 % β)/**61 **(*111101*; 87 % β)).

### Loop cluster analysis

#### Definition of loop clusters

Hydrophobic cluster analysis relies on the marked propensities of hydrophobic residues (VILFMYW) to constitute the internal faces of regular secondary structures. Conversely, a second group of amino acids, constituted by P, G, D, N and S, are clear markers of loops (or coils), as they have higher loop-forming propensities than for the two regular secondary structures [9].

Thus, we aimed at investigating "loop" clusters that are formed by the five residues P, G, D, N and S, using the standard connectivity distance of 4. Loop clusters are defined in the same way that hydrophobic clusters, with PGDNS coded by "1" and any other amino acid coded by "0" (Figure 1). Any loop cluster begins and ends by "1" and no stretch of more than 3 consecutive "0" can be found within its limits. In order to avoid the overlap with regular secondary structures (centered on hydrophobic clusters), we omitted from the primitive PGDNS clusters (gray in Figure 1) information belonging to hydrophobic clusters that would be included in it. However, in this context, we did not consider the hydrophobic clusters *1*and *11*, which are poorly associated with regular secondary structures (see above). The resulting clusters (colored blue in Figure 1) would mostly cover coil positions and were named "loop" clusters. Thus, hydrophobic clusters and loop clusters are not intertwined. Hence, in the example shown in Figure 1 and while using the rule described above, the first defined PGDNS cluster DQRNTLDLIAPSPAD (*100100100011101*), in which the hydrophobic cluster is underlined, is restricted to the DQRN (loop cluster *1001*) and PSPAD (loop cluster *11101*) sequences, with each loop cluster beginning and ending by a «1». The hydrophobic cluster *1*(M at the end of the sequence, Figure 1) is included in the loop cluster SGSMD (*11101*). Mean APC coil values were calculated for the PGDN(S) and for the loop clusters present in the 90 % redundancy database, using a PGDNS alphabet, as well as a reduced PGDN alphabet. The coil preference is maximal for loop clusters (67.0 % coil), relative to PGDNS clusters (51.9 % coil), thus supporting the subtraction of the hydrophobic information. Mean APC coil values are also higher for loop clusters when using the PGDNS alphabet (67.0 % coil) instead of the reduced PGDN alphabet (64.7 % coil).

#### Redundancy treatment

**Figure 6:**
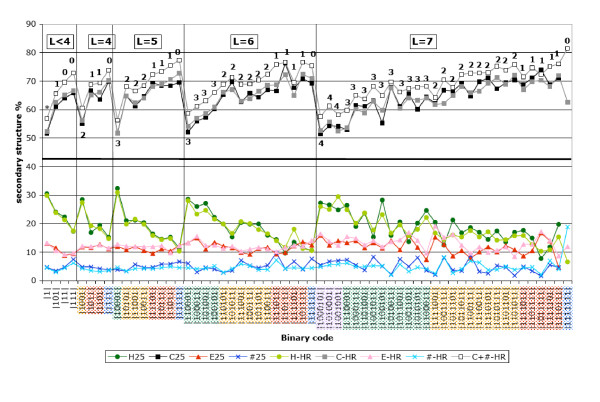
**Percentages of association of loop clusters (length between 1 and 7) with secondary structures**. Frequencies were calculated using the APC rule. The "#' state, which corresponds to missing information in the PDB files (mainly positions for which no electronic density was observed) is also represented and considered with the coil state (c+#). The percentages are indicated for the high redundancy (HR) database (90 %, with the exception of a few clusters for which the chosen redundancy level was lower, Additional file 5), as well as for the 25 % database, when occurences of the considered loop cluster species are sufficient (≥30). Loop clusters are classified, within a given cluster length (L<4, L = 4, L = 5, L = 6 and L = 7), in the decreasing order of the number of residues "0" (this number is indicated near to the coil values, at the top of the figure).

As for hydrophobic clusters, redundancy must be reduced to avoid statistical bias. We adopted the same strategy as for hydrophobic clusters to reduce redundancy (see before). Nine loop clusters species were discarded out of the 176 species populated with at least 30 members at 90% of redundancy, and 15 were considered at lower level of redundancy (lowest level of redundancy 70 %). These levels are indicated in the table reporting the structural preferences of these loop clusters, supplied as Additional file 5 (also see [24])). As for hydrophobic clusters, the observed features of loop cluster species are remarkably stable relative to redundancy (Figure 6). The lengths of sufficiently populated loop cluster species in Additional file 5 vary between 1 and 10, with a maximal number of species for length 8 (51 species). At length 10, only 8 species are represented.

#### Association with coil structures

Only the APC rule was used here to appreciate the general correspondence between loop clusters and observed secondary structures, as we only considered the global coil percentage associated to each loop cluster species. Indeed, the OPS rule, if used, would have associated the 100 % value with nearly all loop clusters. Results with the consensus assignment are detailed below. However, similar results were obtained using other assignment methods (data not shown).

As for hydrophobic clusters towards regular secondary structures, there is a clear preference of loop clusters for coil structures (Additional file 5 and Figure 6). For a given length, the coil frequencies increase with the number of "1" (P, G, D, N or S), the highest ones being observed for "1"-rich loop clusters. This behavior contrasts with that observed for hydrophobic clusters, in which a right balance in "1" and "0" (not too few and not too many) is generally observed [14]. For loop clusters of moderate length (up to 7), the frequencies of association with β-strands are relatively constant (~10 %) whereas the α-helix frequencies vary between 10 and 30 %. This observation illustrates the frequent overflowing of regular secondary structures (especially α-helices) outside of the hydrophobic cluster limits, within the loop cluster borders. Of note is the higher participation of loop clusters exclusively composed of "1" in regions of experimental structures lacking observable electronic density and attributed by current assignment methods as "coils" (loop cluster 127 (*1111111*) in Figure 6).

An overall gain in coil frequencies was observed when loop clusters were considered rather than PDGNS clusters, thus omitting from PDGNS clusters the information provided by hydrophobic clusters (normalized difference above 0 in Additional file 6). This gain is particularly marked for clusters rich in 0, which can include hydrophobic residues making part of hydrophobic clusters and associated regular secondary structures.

### Comparison of secondary structure assignments and PSI-PRED predictions within the hydrophobic cluster limits

**Figure 7:**
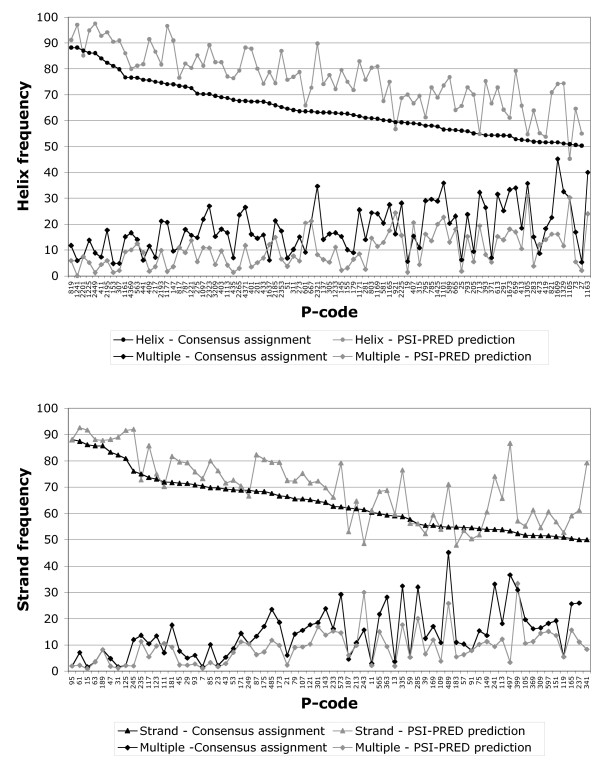
**Comparison of the CONSENSUS assignments made on the limits of hydrophobic clusters typically associated (≥50%) with α-helices or β-strands with prediction performed using PSI-PRED (OPS rule)**. Only the preferred regular secondary structure is represented, as well as the "multiple" assignment. The clusters are classified according in the decreasing order of helix or strand assignment, respectively.

We aimed at comparing the observed structural information contained in hydrophobic clusters, representing "structural words", with predictions made using current secondary structure predictors, such as PSI-PRED [21]. We considered hydrophobic clusters significantly associated with α-helices or β-strands (≥50 % of association with α-helices (87 hydrophobic cluster species) or with β-strands (68 hydrophobic cluster species)) and compared the secondary structure frequencies deduced from observation (consensus) and from prediction (PSI-PRED) (OPS rule) (Figure 7). We observed that the frequencies of association of hydrophobic clusters typical of α-helices with this secondary structure are lower than those arisen from the PSI-PRED prediction (Figure 7, mean: 64.5 % (consensus) versus 76.1 % (PSI-PRED)), indicating that α-helices are over-estimated by PSI-PRED. The same is true, to a lesser extent, for β-strands (Figure 7, mean: 64.3 % (consensus) versus 70.0 % (PSI-PRED)). It is interesting to note that this over-prediction on typical bricks of regular secondary structures is predominantly made at the detriment of the "multiple" assignment, and not of the non-preferred regular secondary structure, as shown in Figure 7 (*helix-forming hydrophobic cluster species: *multiple mean: 18.2 % (consensus) versus 10.0 % (PSI-PRED); *strand-forming hydrophobic cluster species: *multiple mean: 14.5 % (consensus) versus 9.0 % (PSI-PRED)). Over-prediction with PSI-PRED at the detriment of multiple assignment may arise from different situations, as illustrated in Additional file 7. In the case of multiple clusters including only H or E assignments (A), short loops or local irregularities within regular secondary structures, such as kinks or bulges that may lead to interruption of the H or E secondary structure assignment, can not be perceptible by secondary structure predictors. In the case of mixed H/E assignments in multiple clusters (B), the "stronger" regular secondary structure may switch off the signal of the other, weaker, regular secondary structure. Finally, "discordances" can also occur between observation and prediction (C), with an E "multiple" assignment for a single H prediction, as helices are on average twice longer than strands.

## Discussion

Hydrophobic Cluster Analysis (HCA) is generally used in an empirical way to combine secondary structure information with analysis of the primary structure. Indeed, it provides a direct, accurate statistical access to the gravity centers of regular secondary structures through hydrophobic clusters [10,14]. HCA contrasts with other predictive approaches based on hydrophobicity and use of binary patterns (e.g. [25-27]) by providing additional, topological information through the connectivity distance associated with the use of a 2D representation. This information allows evolving from a literal analysis to a lexical one. The so-defined hydrophobic clusters are non-intertwined (they can not include nor to be included in other hydrophobic clusters), and thus correspond to words, which are structurally relevant as they match the positions of regular secondary structures [10]. However, outside the earlier description of the general correspondence of hydrophobic clusters and regular secondary structures, independent of their nature (helices or strands), no detailed analysis has been yet published with respect to the individual secondary structure preferences of each hydrophobic cluster species.

**Figure 8:**
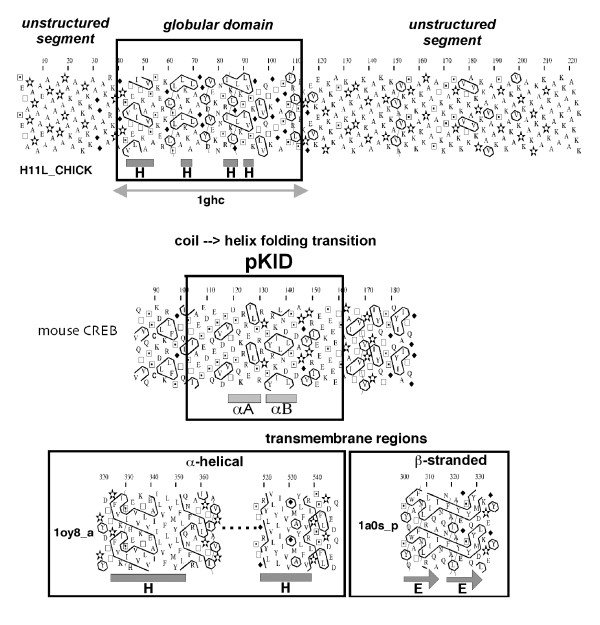
**Prediction of the limits of different protein domains through the HCA plot texture**. ***Top panel***A globular domain, preceded and followed by unstructured segments (histone H1, SW:H11L_CHICK, globular domain pdb 1ghc). The globular domain includes ~33 % of hydrophobic residues (V, I, L, F, M, Y, W), gathered into clusters whose lengths are typical of those of regular secondary structures. In contrast, no or only small hydrophobic clusters are present in the unstructured segments. The observed secondary structures are shown below the plot (H helix). ***Middle panel***Example of an intrinsically disordered segment (phosphorylated kinase-inducible domain (pKID) of mouse CREB), which undergoes a coil --> helix transition upon binding to its partner (KIX domain of the coactivator CBP) [28]. Hydrophobic clusters suggest the presence of regular secondary (α-helices). ***Bottom panel***Segments of membrane proteins, showing the typical texture associated with transmembrane α-helices (*E.coli *acriflavine resistance protein b; pdb 1oy8 (chain a), and transmembrane β-strands (*S.typhimurium *sucrose-specific protein; pdb 1a0s (chain p). A typical example of a HCA-based analysis of membrane proteins can be found in [44] (comparison of E.coli AmtB and human Rh proteins).

Here, we provide a dictionary of 294 hydrophobic clusters frequently encountered in protein globular domains, which allows the estimation of the nature of associated regular secondary structures, from the knowledge of single sequences. This information may help the non-expert user to apply such a methodology. It is important to note that the statistical analysis presented in this manuscript is limited to globular domains of proteins, and does not address the features of transmembrane domains or intrinsically unstructured segments, for which not enough experimental data are generally available at the 3D level. Globular domains of proteins include hydrophobic clusters whose lengths are typical of regular secondary structures, with a mean content in hydrophobic residues of 33 %. This percentage has been calculated for the globular domains included in our databases (SCOP classes A, B, C, D and E, *see Methods*) and, remarkably, is constant whatever the level of redundancy within the databases is (32.63 ± 0.11). In contrast, unstructured regions are generally devoid of, or poor in hydrophobic clusters typical of regular secondary structures. These features are illustrated in Figure 8. It is worth noting that unstructured segments, which may fold upon binding to a partner, generally possess hydrophobic clusters typical of regular secondary structures, which may reveal the potential structural transition. For example, the two helices which were formed in the phosphorylated kinase-inducible domain (pKID) of mouse CREB, upon binding to KIX [28], are associated with two hydrophobic clusters, the second one (P-code 153) showing high preference to α-helices (Figure 8). On the other hand, hydrophobic clusters present in transmembrane domains are different from those typical of globular domains: helical transmembrane segments are generally associated to long hydrophobic clusters, particularly rich in hydrophobic residues, whereas β-stranded membrane proteins, such as porins, possess hydrophobic clusters typical of β-strands, but which are unusually long. These features are also illustrated in Figure 8. Therefore, these "texture" features that can be deciphered through HCA allow an overall appreciation of many structural features at a glance, including the prediction of limits of structured (globular), unstructured and transmembrane domains, which may be refined by automatic methods of prediction. This ability of HCA for unveiling structured/non-structured regions has recently been described in a recent review by Ferron et al. [29]. An original HCA-based method for the prediction of unfolded segments was also recently reported [30], and an HCA-based automatic prediction of globular domains is currently under development in our group.

**Figure 9:**
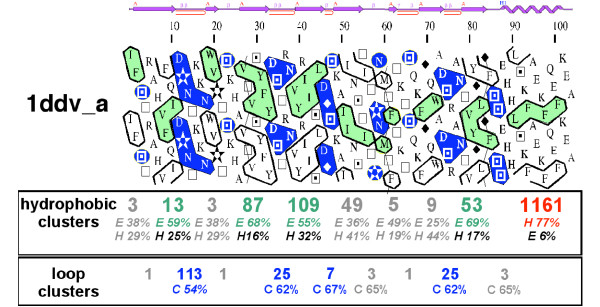
**Generalized cluster analysis applied to a protein sequence**. The sequence of the rat homer evh1 domain (pdb 1ddv, chain A) is shown. Observed secondary structures, as deduced from the experimental three-dimensional structure, are shown above the HCA plot. The P-codes of the hydrophobic and loop clusters are indicated below the HCA plots and are colored when the corresponding clusters have noticeable secondary structure preferences, as reported in Additional files 3 and 5 (green: β-strands, red: α-helices, blue: coil). The corresponding frequencies of association with the preferred secondary structure (OPS and APC rules for the hydrophobic and loop clusters, respectively) are indicated below the P-codes. The small clusters, which have no clear preferences for regular secondary structures, are indicated in grey. Note that the hydrophobic cluster with P-code 49, which is associated at 41 and 36 % with α-helices and β-strands, respectively, can be suggested in a β-strand conformation, owing to its amino acid composition (three isoleucine, one threonine).

In order to provide additional and accurate information on non-regular secondary structures, we also propose in this report an analysis of loop clusters. Loop clusters are built with the PGDNS alphabet, using the same connectivity distance than hydrophobic clusters (4) and without overlap with hydrophobic clusters (Figure 1). The use of a loop alphabet for the construction of clusters was already shown to give the minimal scores of association with regular secondary structures [10]. Here, we show that loop clusters are associated at high level with coil structures and that the coil association frequencies are closely connected with the number of P,G,D,N,S residues. In contrast, hydrophobic clusters possess not too few but also not too many hydrophobic amino acids to carry relevant secondary structure information [14]. Thus, adding "loop cluster analysis" to "hydrophobic cluster analysis" into a "generalized cluster analysis" allows the coverage of a large fraction of protein sequences, giving accurate information on regular as well as non-regular secondary structures. An example of "GCA" analysis that can be performed is given in Figure 9.

**Figure 10:**
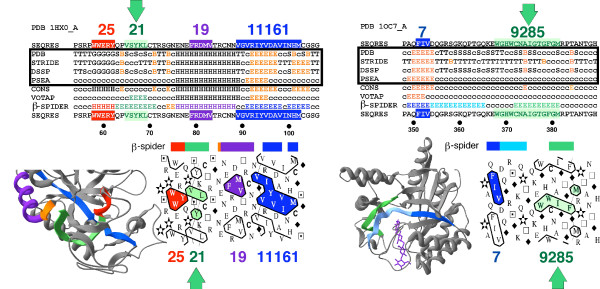
**Two examples of hydrophobic clusters assigned as "coil" using the consensus method and in an extended conformation using both VoTAP and β-SPIDER (left) and β-SPIDER alone (right)**. The alternative assignments of these regions by β-SPIDER were originally described in [35]. The two structures correspond to: at left, the pig pancreatic alpha-amylase (pdb 1h×0, chain A) and at right, a mutant of the cellobiohydrolase cel6a from *Humicola insolens *in complex with methyl-tetrathio-alpha-d-cellopentoside (pdb 1oc7, chain A). Results of the different assignment methods were shown at top, together with the 1D sequence, in which hydrophobic clusters and associated Peitsch codes are colored. Hydrophobic clusters are reported accordingly on the HCA plots. The coloring scheme adopted for the β-SPIDER assignment is reported on the 3D level.

The quality of results presented in this study obviously depends on the quality of secondary structure assignments, which generally show some differences in the secondary structure limits [5]. In an exhaustive approach, we have considered three different assignment methods (DSSP [31], STRIDE [32] and PSEA [33]), in addition to the expert-based assignments found in the PDB files. A consensus assignment was also built from the outputs of these four different methods and was used in particular in this study. All methods gave similar results relative to the observed percentages of cluster association with secondary structures. However, new assignment methods, such as VoTAP [34] or β-SPIDER (dedicated to an efficient assignment of extended structures [35]) may be integrated in the future in such a study. These alternative methods can indeed be useful to further investigate the rare hydrophobic clusters (outside the small basic hydrophobic clusters 1, 3, 5, 9 and 17) associated with coils (as defined using DSSP or the consensus assignment) and which, in fact, can be associated with regular secondary structures, especially extended ones. Figure 10 illustrates this observation with two cases of hydrophobic clusters associated with coil structures (as defined using the consensus assignment), whereas both VoTAP and β-SPIDER, or β-SPIDER alone, assigned extended structures (P-codes 21 and 9285, respectively). Hydrophobic cluster with P-code 21, which is included in our dictionary, has a shape typical of β-strand (66 % in extended structure using the OPS rule), whereas the shape of the uncommon hydrophobic cluster with P-code 9285, absent from our dictionary, is typical of long and mobile extended structures (data not shown). It would be interesting to further analyze the amino acid composition of these few hydrophobic clusters assigned in the coil state.

In a more general way, this analysis of amino acid composition would also be a critical point for accurately predicting the nature of secondary structures associated with the different hydrophobic cluster species. Amino acid profiles, calculated for each of two regular secondary structure (α and β) associated to each cluster species should allow the refinement of the secondary structure prediction. This however requires solving to a better extent the problem raised by multiple clusters, that is clusters which are associated with at least two regular secondary structures (see below).

Information about amino acid composition would also be useful for refining the analysis of truncated hydrophobic clusters that only cover a limited part of the associated regular secondary structure. Examples of "truncated" hydrophobic clusters can be found in Figure 1 (P-code 403) and Figure 10 (P-code 19). Amino acids in the vicinity of these hydrophobic clusters, such as A, C and T that can substitute for strong hydrophobic residues, might indicate the overflowing of the hydrophobic cluster by the associated regular secondary structure.

Within families of proteins, hydrophobic clusters rapidly evolve relative to sequence divergence, around a stable core of "topohydrophobic" residues (hydrophobic residues which occupy, in a multiple alignment, positions that are always substituted by hydrophobic amino acids and constitute the core of globular domains) [36,37]. In this context, an interesting perspective to help the HCA-based comparison of divergent sequences is to analyze the cluster substitution schemes within families of sequences, around the invariable kernel of topohydrophobic residues. The so-defined cluster substitution matrices might thus constitute sensitive tools to identify structural conservation at low levels of sequence identity. Alternatively, "canonical" clusters, which show clear preferences for α or β secondary structures, can orientate in a recursive way the prediction for other less typical clusters, with which they are aligned within a family of proteins.

Noticeably, characteristics deduced from the analysis of hydrophobic cluster species appear quite stable relative to divergence, as illustrated in this study by several variables analyzed at different levels of redundancy (secondary structure association frequencies, cluster propensities, amino acid compositions). The same behavior is observed with loop clusters. As a consequence, high redundancy databases can be exploited in order to get a high number of statistically valuable clusters, provided that artifacts are eliminated by cluster species (*see Results section*). Information can be gained about the majority of strands and some helices (cluster lengths up to 13 residues). This size limitation will be progressively overcome by the continuous increase in experimental three-dimensional structures reported in the Protein Data Bank, even though new entries are related to already known structures, owing to this non-limitation relative to low redundancy levels. A specific handling of long hydrophobic clusters might also allow the separation of long clusters, which are often "multiple" (*that is*, covering at least two regular secondary structures but with loops of length inferior to the considered connectivity distance), into their single components and, as a consequence, make them available for prediction, as suggested by recent results. This procedure would consider clusters of PGNDS residues included in hydrophobic clusters (thus opposite to loop clusters), which often underline the presence of secondary structure separators into these multiple clusters. At the same time, the HCA formalism can be adjusted to the context to improve predictions. Depending on cluster species, different helical pitches (*i.e*. connectivity distance or CD) can indeed be considered ([14]) to optimize the discrimination power. For particular clusters, one isolated hydrophobic residue, located in the N-ter or C-ter, may artificially lengthen it, leading to a biased prediction of the secondary structure limits (*e.g*. in the sequence DPKKINTRFLLYTNENQ, the two first positions of the hydrophobic cluster, which is underlined, are assigned as coil). As a consequence, a lower connectivity distance (CD) would be more appropriate for such clusters. Accordingly, one can observe, for example, that the hydrophobic cluster species ***10011001***has a slightly higher discrimination power with a CD of 3 rather than 4. In contrast, ***110101***shows an optimum for a CD of 5. Considering systematically lower CD however leads to the artifact increase of helix-associated clusters of small length, and to an overall decrease of the structural two-state (regular secondary structure/coil) overlap between hydrophobic clusters and regular secondary structures. On another hand, higher CD generally leads to the artifact increase of the number of multiple clusters. CD4 appears to be the best compromise between discrimination power and artifact minimization. Otherwise, symmetric clusters (*e.g*. *1011and 1101*) typically show similar behavior relative to association with secondary structures, as shown in the Results. Thus, for sparsely populated clusters, the addition of symmetric cluster data may be an interesting way to expand the set of useful clusters, in particular for high cluster lengths.

## Conclusion

In combination with loop clusters, which were here analyzed for the first time, hydrophobic clusters may provide useful and accurate information about secondary structures of a large part of globular protein domains, from the only knowledge of a single sequence. The dictionary of hydrophobic and loop cluster presented here may help the user to apply such a methodology. The analysis deduced form single sequences could of course be improved by considering multiple alignments of sequences belonging to a same family, when possible. Indeed, some hydrophobic clusters, which are not very informative or for which statistics are lacking, can be substituted in other sequences of the family by much more informative clusters, allowing to refine the HCA-based prediction. HCA can thus be used in an iterative and synergetic way with other efficient and automatic methods, in order to improve prediction taking into account multiple alignments. Finally, the general statistics described here, relative to the structural preferences of hydrophobic and loop clusters and to their structure-dependent composition in amino acids, could be used to design a predictive tool, which might be integrated in automatic procedures for comparing highly divergent sequences.

## Materials and methods

### Databases

We considered the SCOP database (version 1.69, July 2005) [38,39], in which we selected proteins of the first five classes (all alpha, all beta, alpha/beta, alpha+beta, multidomains). From this set (23571 PDB files, corresponding to 49068 protein chains), we discarded protein chains of the first five classes, which are also reported in other SCOP classes. We only kept X-ray structures, and discarded protein models and obsolete entries, following the classification available from the Research Collaboratory for Structural Bioinformatics (RCSB) database [40], as well as files containing only Cα coordinates. Our final database contains 46228 protein chains. Then, we used the PISCES server [41] for culling protein chains from this list by sequence identity (ranging from 5% to 95% identity, by step of 5 %). The different databases obtained in this way contain from 1815 (5%) to 7015 (95 %) protein chains. The databases specifically used in this study contain 2925 (25%) and 6663 (90 %) protein chains.

### Secondary structure assignments

We used different methods for assigning secondary structures from atomic coordinates, including DSSP [31], STRIDE [32] and PSEA [33]. Secondary structures defined in the PDB files (SS_PDB) were also considered. A consensus assignment was deduced from the consideration of these four methods. A standard reduction of the different secondary structure states produced as outputs by DSSP (8 states) and STRIDE (7 states) to three states (helix (H), strand (E) and default coil (C)) was first performed using the "EVA" rule conversion ([3,42]). In this scheme, α-helix (H), 3_10 _helix (G) and π-helix (I) are converted to H, extended (E) and isolated β-bridge (B) to E and turn (T), bend (S) and other to C. The consensus assignment was then obtained by indicating the most frequent secondary structure state among the DSSP, STRIDE, PSEA and SS_PDB assignments. If two secondary structures arise with the same frequency, the regular secondary structure is preferred over coil. If a helix assignment competes with a strand one, the corresponding position is assigned as coil.

### Association of clusters with secondary structures

Two rules can be defined to measure the association of hydrophobic clusters with secondary structures. The **APC rule **(***all positions considered***) takes into account the assignments linked to all positions of a given cluster species and can be calculated as:

APCs(X)=∑n=1NxnsNxLx
 MathType@MTEF@5@5@+=feaafiart1ev1aaatCvAUfKttLearuWrP9MDH5MBPbIqV92AaeXatLxBI9gBaebbnrfifHhDYfgasaacH8akY=wiFfYdH8Gipec8Eeeu0xXdbba9frFj0=OqFfea0dXdd9vqai=hGuQ8kuc9pgc9s8qqaq=dirpe0xb9q8qiLsFr0=vr0=vr0dc8meaabaqaciaacaGaaeqabaqabeGadaaakeaacqqGbbqqcqqGqbaucqqGdbWqdaWgaaWcbaGaee4CamhabeaakiabcIcaOiabbIfayjabcMcaPiabg2da9maalaaabaWaaabCaeaacqqGUbGBdaWgaaWcbaGaee4CamhabeaaaeaacqqGUbGBcqGH9aqpcqaIXaqmaeaacqqGobGtdaWgaaadbaGaeeiEaGhabeaaa0GaeyyeIuoaaOqaaiabb6eaonaaBaaaleaacqqG4baEaeqaaOGaeeitaW0aaSbaaSqaaiabbIha4bqabaaaaaaa@467A@

where s is the secondary structure assignment (H, E or C), n_s _is the number of "s" assignment in a hydrophobic cluster of species X, N_x _is the occurrence of hydrophobic clusters within the species X and L_x _is the length of the hydrophobic cluster species X.

Another way to evaluate the association of hydrophobic clusters with secondary structures relies on the **OPS rule **(***one position is sufficient***). According to this rule, the entire cluster is assumed to be associated with a helix or with a strand if, within this cluster, one or several amino acids are assigned in the helix or strand state, respectively. Clusters which contain both α and β assignments or which contain two strings of regular secondary structures separated by coil positions are considered apart and are called multiple. The frequency of association with the helix, strand or multiple states with respect to the OPS rule can be calculated as follows:

OPSs(X)=nXsNx
 MathType@MTEF@5@5@+=feaafiart1ev1aaatCvAUfKttLearuWrP9MDH5MBPbIqV92AaeXatLxBI9gBaebbnrfifHhDYfgasaacH8akY=wiFfYdH8Gipec8Eeeu0xXdbba9frFj0=OqFfea0dXdd9vqai=hGuQ8kuc9pgc9s8qqaq=dirpe0xb9q8qiLsFr0=vr0=vr0dc8meaabaqaciaacaGaaeqabaqabeGadaaakeaacqqGpbWtcqqGqbaucqqGtbWudaWgaaWcbaGaee4CamhabeaakiabcIcaOiabbIfayjabcMcaPiabg2da9maalaaabaGaeeOBa4MaeeiwaG1aaSbaaSqaaiabbohaZbqabaaakeaacqqGobGtdaWgaaWcbaGaeeiEaGhabeaaaaaaaa@3CCA@

where nX_s _is the number of hydrophobic clusters assigned as "s" following the OPS rule and N_x _the total number of hydrophobic cluster within the species X.

Coverage of the hydrophobic cluster species assigned in the helix and strand state following the OPS rule by the corresponding secondary structures is calculated as follows:

covs(X)=∑n=1MmsnXsLx
 MathType@MTEF@5@5@+=feaafiart1ev1aaatCvAUfKttLearuWrP9MDH5MBPbIqV92AaeXatLxBI9gBaebbnrfifHhDYfgasaacH8akY=wiFfYdH8Gipec8Eeeu0xXdbba9frFj0=OqFfea0dXdd9vqai=hGuQ8kuc9pgc9s8qqaq=dirpe0xb9q8qiLsFr0=vr0=vr0dc8meaabaqaciaacaGaaeqabaqabeGadaaakeaacqqGJbWycqqGVbWBcqqG2bGDdaWgaaWcbaGaee4CamhabeaakiabcIcaOiabbIfayjabcMcaPiabg2da9maalaaabaWaaabCaeaacqqGTbqBdaWgaaWcbaGaee4CamhabeaaaeaacqqGUbGBcqGH9aqpcqaIXaqmaeaacqqGnbqta0GaeyyeIuoaaOqaaiabb6gaUjabbIfaynaaBaaaleaacqqGZbWCaeqaaOGaeeitaW0aaSbaaSqaaiabbIha4bqabaaaaaaa@4727@

where m_s _is the number of "s" assignments in a hydrophobic cluster of species X assigned as "s" by the OPS rule, nX_S _is the number of hydrophobic clusters of species X assigned as "s" following the OPS rule, and L_X _the length of the hydrophobic cluster of species X.

These values were calculated for all redundancy levels (by steps of 5 %).

### Cluster propensities

Propensities of hydrophobic clusters for a secondary structure state (C, E, H or M) were calculated in the same way than amino acid propensities. Cluster propensities are calculated as follows:

^S^P_X _= ^S^F_X_/F_X_,

where ^S^F_X _= (nX_S_/nS) (^S^F_X _is the frequency of the hydrophobic cluster species X in the state "s", nX_S _is the number of the hydrophobic clusters of species X in the state "s" (OPS rule), nS is the total number of hydrophobic clusters in the state "s" (OPS rule)) and F_X _= (nX/N) (F_X _is the frequency of the hydrophobic cluster of species X, nX is total number of hydrophobic clusters of species X and N is the total number of hydrophobic clusters).

### Secondary structure predictions

Secondary structures of the different sequences reported in our structure databases were predicted using PSI-PRED [21].

### HCA plots

HCA plots were drawn using the DrawHCA server [43].

## Authors' contributions

RE and KLT participated in the design of the study and carried out the statistical analysis of hydrophobic and loop clusters. JD participated in the coordination of the study, together with JPM and IC who conceived it and drafted the manuscript. All authors read and approved the final manuscript.

## Supplementary Material

Additional File 1 **Distribution of hydrophobic cluster lengths (species populated with at least 30 members) within banks at different level of sequence redundancy (5 %, 25 %, 50 %, 90 %)**. Bars indicate the number of hydrophobic cluster species of a given length, for which cluster occurrence is equal or greater than 30. The sum of the 5% bars (from length 1 to 12) is equal to 97. Click here for file

Additional File 2 **Hydrophobic cluster occurrences within each hydrophobic cluster species, at different levels of redundancy**. ***Top panel***Occurrences were normalized by values at 50 % of redundancy (light blue straight line at 1.00). 50 % was chosen as a reference, as it roughly corresponds to the inflection points of the curves reporting, for each cluster species, cluster occurrences as a function of the redundancy level (see below). Moreover, it is halfway between the two extreme values of redundancy (5% and 95%). This figure illustrates a representative sample of 20 species, out of the 304 species populated with at least 30 members at 90 % of redundancy. *Bottom panel *Occurrences at different levels of redundancy, illustrated for the species 153 (blue arrow) and 137 (pink arrow). Click here for file

Additional File 3 **Hydrophobic clusters analysis: structural features of 294 hydrophobic cluster species, which are frequently encountered in protein globular domains**. The considered redundancy levels and the occurrences within each hydrophobic cluster species are indicated. Frequencies of association with secondary structures (CONSENSUS assignment) are given according to the APC and OPS rules (see text), and the associated α- and β-coverage values are reported, together with the hydrophobic cluster propensities. The affinity of a hydrophobic cluster species towards a secondary structure is defined by the maximal propensity. High affinities (capitals: H, E, C or M) are assigned if other propensities are lower than 1 or if the difference between the highest propensity value and the second one is greater than 1. Maximal frequency and propensity values are shaded (several frequency values are shaded if they don't differ of more than 15 units). This table can also be found on our Web server [23]. Click here for file

Additional File 4 **Stable features of hydrophobic clusters relative to redundancy**. The frequencies of association of two hydrophobic cluster species, typical of α-helices (P-code 153, *10011001*) and β-strands (P-code 15, *1111*) with secondary structures, determined using the OPS rule, were reported at the different levels of redundancy. Click here for file

Additional File 5 **Loop clusters analysis. Structural features of 167 loop cluster species, which are frequently encountered in protein globular domains**. The considered redundancy levels and the occurrences within each loop cluster species are indicated. Frequencies of association with secondary structures (CONSENSUS assignment) are given according to the APC rule (see text). # indicates a position for which no coordinate has been reported in the analyzed PDB files. This table can also be found on our Web server [24]. Click here for file

Additional File 6 **Normalized differences of the percentages of association with secondary structures (α,β, coil) between loop clusters and PGDNS clusters ((%_state_loop - %_state_PDGNS)/-%_state_PDGNS) × 100)**. These differences were calculated on the basis of the 25 % database and of the consensus assignment. Click here for file

Additional File 7 Three examples of PSI-PRED overprediction of helices, at the detriment of the multiple assignment (Consensus line). Click here for file
