# Supplementary figures and images for: A generalized analysis of hydrophobic and loop clusters within globular protein sequences
Source: BMC Struct Biol. 2007 Jan 8;7:2. doi: 10.1186/1472-6807-7-2 (PMC1774571; doi:10.1186/1472-6807-7-2)

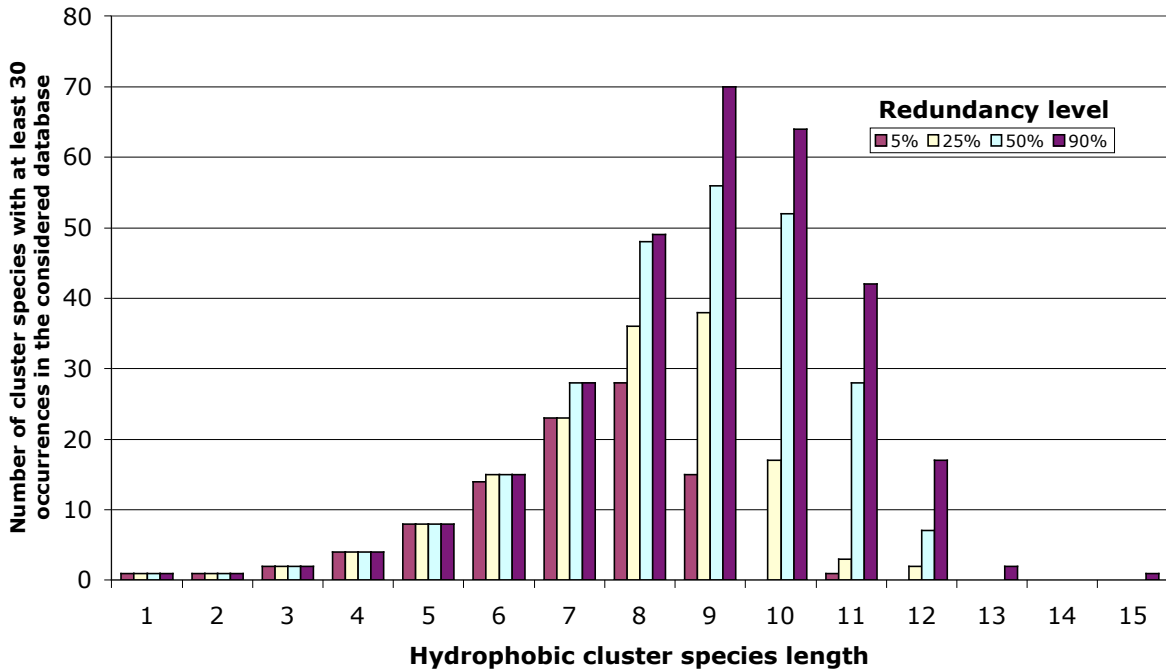

Supplement: Additional File 1 — Distribution of hydrophobic cluster lengths (species populated with at least 30 members) within banks at different level of sequence redundancy (5 %, 25 %, 50 %, 90 %). Bars indicate the number of hydrophobic cluster species of a given length, for which cluster occurrence is equal or greater than 30. The sum of the 5% bars (from length 1 to 12) is equal to 97. [file 1472-6807-7-2-S1.pdf]

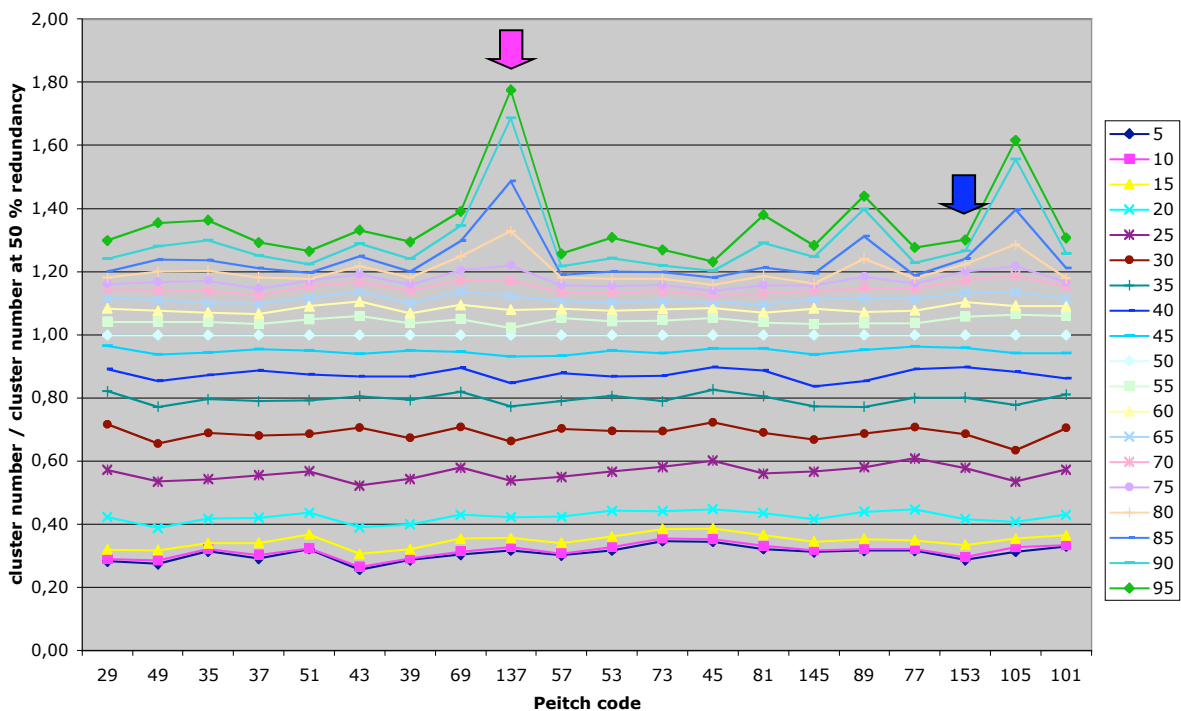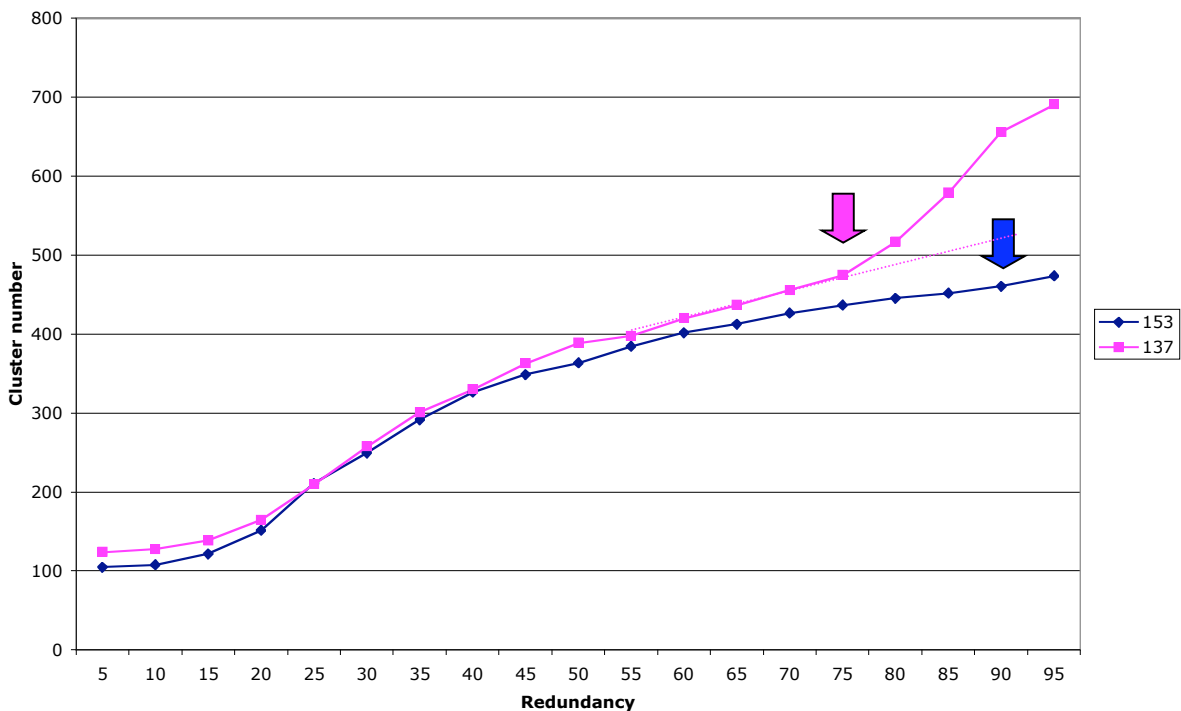

Supplement: Additional File 2 — Hydrophobic cluster occurrences within each hydrophobic cluster species, at different levels of redundancy. Top panelOccurrences were normalized by values at 50 % of redundancy (light blue straight line at 1.00). 50 % was chosen as a reference, as it roughly corresponds to the inflection points of the curves reporting, for each cluster species, cluster occurrences as a function of the redundancy level (see below). Moreover, it is halfway between the two extreme values of redundancy (5% and 95%). This figure illustrates a representative sample of 20 species, out of the 304 species populated with at least 30 members at 90 % of redundancy. Bottom panel Occurrences at different levels of redundancy, illustrated for the species 153 (blue arrow) and 137 (pink arrow). [file 1472-6807-7-2-S2.pdf]

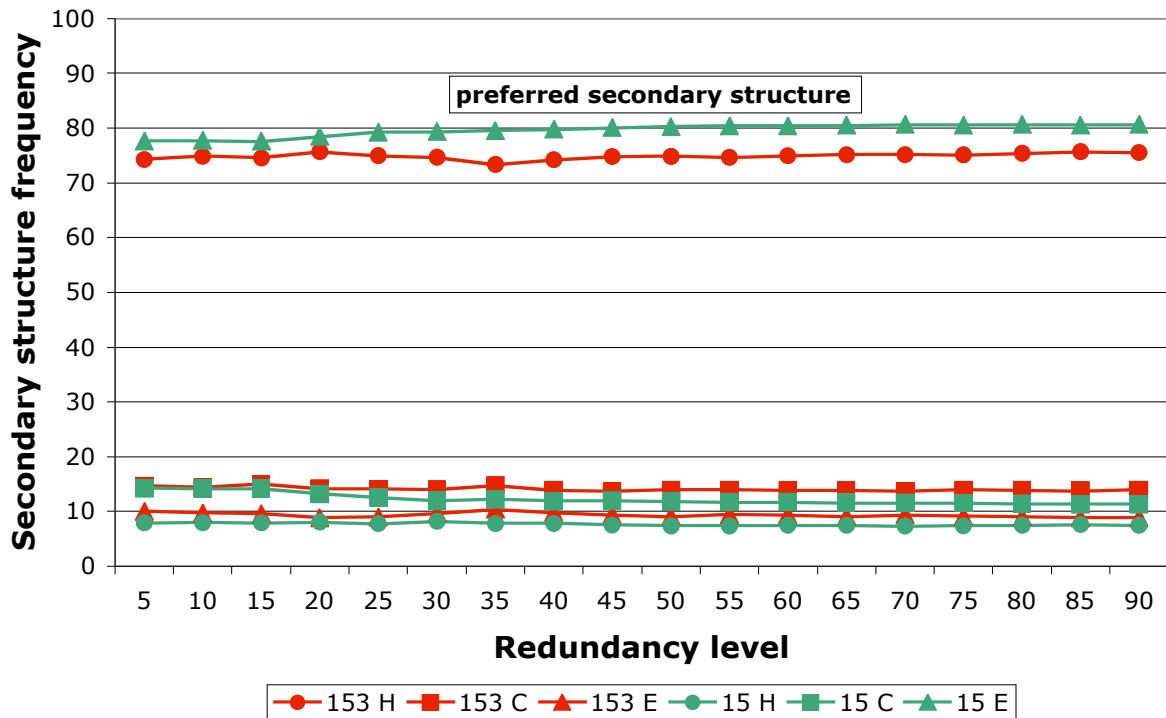

Supplement: Additional File 4 — Stable features of hydrophobic clusters relative to redundancy. The frequencies of association of two hydrophobic cluster species, typical of α-helices (P-code 153, 10011001) and β-strands (P-code 15, 1111) with secondary structures, determined using the OPS rule, were reported at the different levels of redundancy. [file 1472-6807-7-2-S4.pdf]

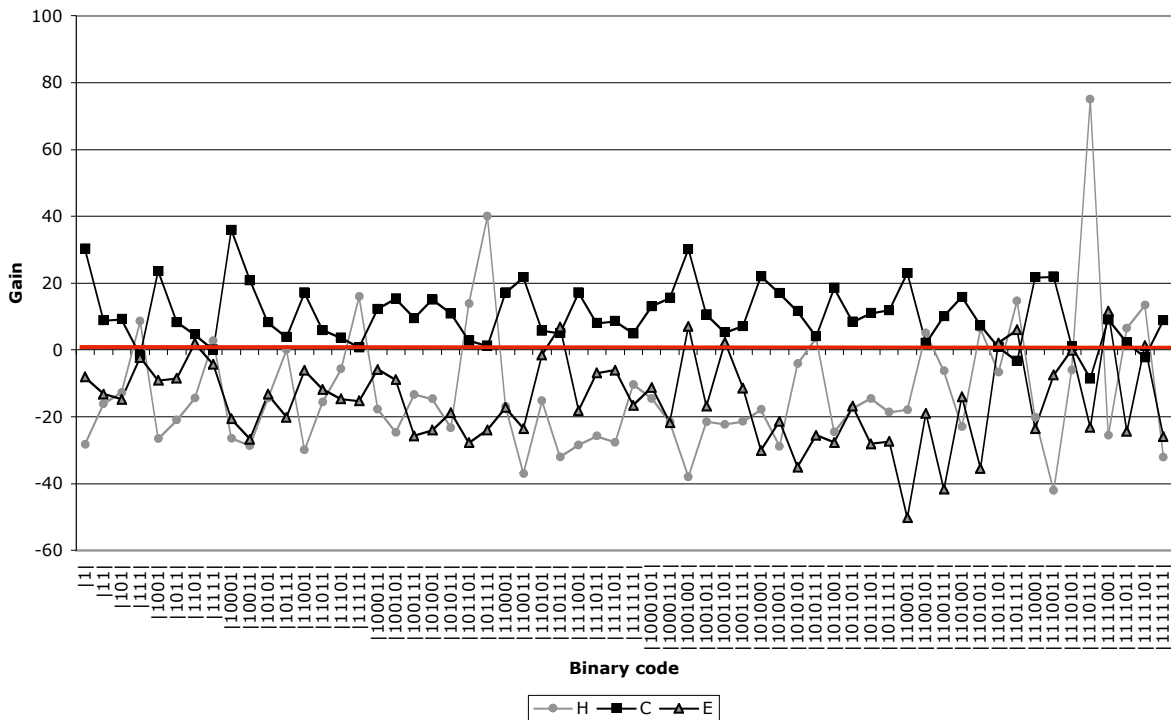

Supplement: Additional File 6 — Normalized differences of the percentages of association with secondary structures (α,β, coil) between loop clusters and PGDNS clusters ((%stateloop - %statePDGNS)/-%statePDGNS) × 100). These differences were calculated on the basis of the 25 % database and of the consensus assignment. [file 1472-6807-7-2-S6.pdf]
