# Supplementary material for: A generalized analysis of hydrophobic and loop clusters within globular protein sequences
Source: BMC Struct Biol. 2007 Jan 8;7:2. doi: 10.1186/1472-6807-7-2 (PMC1774571; doi:10.1186/1472-6807-7-2)
Supplement: Additional File 3 — Hydrophobic clusters analysis: structural features of 294 hydrophobic cluster species, which are frequently encountered in protein globular domains. The considered redundancy levels and the occurrences within each hydrophobic cluster species are indicated. Frequencies of association with secondary structures (CONSENSUS assignment) are given according to the APC and OPS rules (see text), and the associated α- and β-coverage values are reported, together with the hydrophobic cluster propensities. The affinity of a hydrophobic cluster species towards a secondary structure is defined by the maximal propensity. High affinities (capitals: H, E, C or M) are assigned if other propensities are lower than 1 or if the difference between the highest propensity value and the second one is greater than 1. Maximal frequency and propensity values are shaded (several frequency values are shaded if they don't differ of more than 15 units). This table can also be found on our Web server [23]. [file 1472-6807-7-2-S3.pdf]

HYDROPHOBIC CLUSTER		redondancy	number	frequency														
				APC rule			OPS rule				coverage (OPS rule)		propensities (OPS rule)				affinity
P- code	binary code			C	E	H	C	E	H	M	E	H	C	E	H	M	
1	|1|	90	35079	63	17	20	63	17	20	0	100	100	2,80	0,53	0,68	0,00	C
3	|11|	90	12910	40	33	27	32	38	29	1	86	91	1,42	1,19	1,01	0,04	c
5	|101|	85	7851	45	40	15	30	49	19	2	81	76	1,34	1,55	0,65	0,11	e
7	|111|	90	5118	23	63	14	12	70	16	2	89	88	0,55	2,20	0,54	0,10	E
9	|1001|	90	4576	45	18	37	27	25	44	3	67	82	1,21	0,79	1,52	0,19	h
11	|1011|	85	2998	27	51	23	12	60	25	3	82	87	0,51	1,93	0,85	0,18	E
13	|1101|	90	3007	28	51	21	13	59	25	4	84	80	0,56	1,84	0,85	0,23	E
15	|1111|	90	1847	12	81	7	4	86	8	2	93	88	0,19	2,69	0,27	0,10	E
17	|10001|	80	2633	41	18	40	22	25	46	7	63	83	0,95	0,81	1,54	0,45	H
19	|10011|	90	2101	26	19	55	10	25	59	6	68	90	0,45	0,79	2,03	0,34	H
21	|10101|	80	1887	29	57	14	11	66	16	6	82	77	0,49	2,15	0,54	0,37	E
23	|10111|	90	1177	17	61	23	5	70	24	2	86	94	0,20	2,18	0,81	0,13	E
25	|11001|	85	2143	26	24	51	10	28	56	6	76	87	0,43	0,89	1,90	0,38	H
27	|11011|	90	1191	17	35	47	5	39	50	5	84	91	0,23	1,23	1,73	0,32	h
29	|11101|	90	1079	18	65	17	5	71	19	5	87	88	0,21	2,23	0,65	0,31	E
31	|11111|	90	416	10	78	13	2	82	14	2	93	93	0,11	2,57	0,47	0,10	E
35	|100011|	90	1019	36	27	37	14	35	39	12	66	82	0,60	1,11	1,33	0,75	h
37	|100101|	90	1002	42	36	23	13	46	28	12	67	69	0,58	1,44	0,98	0,76	E
39	|100111|	90	698	24	47	29	4	55	29	12	74	88	0,16	1,73	0,98	0,77	E
41	|101001|	90	1086	39	37	24	12	46	29	13	70	69	0,55	1,44	0,98	0,79	E
43	|101011|	90	752	24	58	18	5	69	20	5	81	79	0,22	2,17	0,70	0,33	E
45	|101101|	90	627	25	61	14	5	71	15	8	80	78	0,24	2,23	0,53	0,47	E
47	|101111|	90	311	16	73	11	1	83	11	5	85	85	0,06	2,60	0,36	0,30	E
49	|110001|	90	1074	37	28	35	11	36	41	12	64	79	0,51	1,13	1,39	0,72	h
51	|110011|	90	786	20	18	62	5	23	65	7	68	92	0,24	0,72	2,22	0,42	H
53	|110101|	90	639	24	61	16	5	69	17	9	82	79	0,23	2,16	0,59	0,53	E
55	|110111|	90	251	12	47	41	4	49	43	5	90	94	0,18	1,52	1,46	0,29	e
57	|111001|	90	648	28	45	27	6	55	29	10	73	86	0,25	1,71	1,01	0,63	e
59	|111011|	90	338	15	50	35	2	58	35	5	83	95	0,08	1,80	1,21	0,33	e
61	|111101|	90	310	15	80	5	1	87	5	7	87	80	0,04	2,73	0,15	0,44	E
63	|111111|	90	84	7	82	11	1	86	10	4	93	100	0,05	2,68	0,33	0,22	E
69	|1000101|	90	689	41	27	31	12	35	36	17	58	75	0,51	1,09	1,24	1,07	h
71	|1000111|	90	306	39	39	21	9	48	21	22	63	77	0,41	1,51	0,72	1,32	e
73	|1001001|	90	628	37	18	46	11	22	51	17	53	79	0,47	0,69	1,74	1,04	h
75	|1001011|	90	293	30	44	25	6	54	25	15	72	82	0,26	1,70	0,84	0,94	E
77	|1001101|	90	488	27	34	39	5	41	41	12	72	84	0,22	1,29	1,42	0,76	h
79	|1001111|	90	177	22	59	20	1	66	19	14	77	90	0,05	2,05	0,66	0,87	E
81	|1010001|	85	545	41	30	29	12	39	32	17	61	77	0,53	1,25	1,09	1,01	e
83	|1010011|	90	379	31	30	40	7	32	38	23	72	78	0,29	1,00	1,31	1,43	m
85	|1010101|	85	364	28	59	13	6	70	14	10	79	72	0,25	2,23	0,48	0,62	E
87	|1010111|	90	158	19	64	17	3	68	16	13	86	86	0,11	2,14	0,54	0,82	E
89	|1011001|	80	442	23	42	34	6	50	35	10	78	90	0,26	1,61	1,16	0,59	e
91	|1011011|	75	165	21	48	31	4	55	33	8	80	87	0,18	1,79	1,11	0,48	e
93	|1011101|	90	182	19	60	21	1	71	22	6	82	85	0,05	2,22	0,75	0,37	E
95	|1011111|	90	50	9	81	10	0	88	10	2	90	100	0,00	2,75	0,34	0,12	E
99	|1100011|	90	298	36	34	30	7	36	31	27	67	76	0,30	1,12	1,06	1,63	m
101	|1100101|	90	440	31	33	36	4	38	42	17	70	75	0,18	1,17	1,44	1,02	h
103	|1100111|	90	174	19	42	39	3	44	37	16	81	88	0,13	1,38	1,28	0,95	e
105	|1101001|	80	377	37	42	21	6	52	23	20	67	65	0,25	1,68	0,76	1,20	e
107	|1101011|	85	206	23	61	16	4	66	15	16	83	76	0,17	2,09	0,51	0,95	E
109	|1101101|	90	211	21	50	30	2	55	32	11	82	83	0,08	1,72	1,11	0,67	e
111	|1101111|	90	57	16	67	17	2	72	19	7	85	87	0,08	2,25	0,66	0,43	E
113	|1110001|	90	353	35	44	21	6	54	22	18	67	75	0,25	1,68	0,77	1,11	e
115	|1110011|	90	217	21	42	37	3	47	34	17	77	90	0,12	1,45	1,15	1,05	e
117	|1110101|	90	182	20	68	12	3	74	13	10	85	83	0,15	2,30	0,43	0,64	E
119	|1110111|	90	55	12	46	41	2	51	42	5	87	94	0,08	1,59	1,44	0,33	e
121	|1111001|	90	204	31	55	14	3	65	14	18	69	81	0,13	2,04	0,49	1,08	e
123	|1111011|	90	52	17	68	15	0	73	13	13	85	92	0,00	2,28	0,46	0,83	E
125	|1111101|	90	47	12	71	16	0	81	17	2	86	95	0,00	2,53	0,58	0,13	E

HYDROPHOBIC CLUSTER		redondancy	number	frequency									propensities (OPS rule)				affinity
				APC rule			OPS rule				coverage (OPS rule)						
P- code	binary code			C	E	H	C	E	H	M	E	H	C	E	H	M	
137	|10001001|	75	475	30	11	59	9	14	63	14	49	87	0,39	0,45	2,10	0,86	H
139	|10001011|	90	308	30	27	43	5	36	43	15	58	87	0,23	1,14	1,48	0,94	h
141	|10001101|	90	269	36	36	28	11	37	30	22	67	81	0,48	1,16	1,03	1,35	m
143	|10001111|	90	109	29	58	13	2	64	10	24	70	92	0,08	2,01	0,35	1,46	e
145	|10010001|	90	505	29	12	60	9	13	63	15	57	87	0,41	0,40	2,15	0,94	H
147	|10010011|	90	366	20	10	70	4	12	74	10	58	89	0,18	0,38	2,54	0,59	H
149	|10010101|	90	213	31	46	23	7	54	26	14	74	77	0,29	1,69	0,89	0,84	E
151	|10010111|	90	125	23	47	30	2	51	27	19	75	85	0,11	1,60	0,93	1,18	e
153	|10011001|	90	461	16	9	75	3	11	81	5	66	91	0,13	0,35	2,78	0,29	H
155	|10011011|	85	228	16	20	64	1	26	63	10	65	96	0,06	0,83	2,12	0,62	H
157	|10011101|	90	146	17	45	38	3	47	37	14	81	94	0,12	1,46	1,27	0,84	e
159	|10011111|	75	52	16	42	42	0	48	42	10	78	91	0,00	1,57	1,41	0,59	e
163	|10100011|	90	214	33	34	33	8	29	31	31	66	87	0,37	0,91	1,07	1,92	m
165	|10100101|	90	230	36	50	14	11	50	13	26	75	68	0,48	1,58	0,45	1,58	E
167	|10100111|	90	130	29	44	26	2	39	22	36	68	83	0,10	1,23	0,77	2,22	m
169	|10101001|	80	276	29	47	24	4	55	24	17	69	84	0,16	1,80	0,80	1,04	e
171	|10101011|	90	125	25	62	13	3	69	14	14	79	84	0,14	2,15	0,47	0,88	E
173	|10101101|	90	102	26	60	13	2	67	13	19	75	83	0,09	2,08	0,44	1,14	e
175	|10101111|	90	41	20	66	14	0	68	15	17	83	75	0,00	2,13	0,50	1,05	E
177	|10110001|	90	256	27	33	40	4	37	41	18	65	89	0,17	1,16	1,39	1,13	h
179	|10110011|	75	156	14	24	62	3	26	62	9	80	94	0,11	0,86	2,07	0,55	H
181	|10110101|	90	131	27	63	10	2	72	9	18	75	63	0,07	2,24	0,31	1,08	E
183	|10110111|	90	73	12	50	38	0	55	34	11	86	96	0,00	1,71	1,18	0,67	e
185	|10111001|	90	163	18	40	42	2	50	42	6	76	94	0,11	1,55	1,45	0,34	e
187	|10111011|	90	66	16	50	34	2	72	19	7	85	87	0,08	2,25	0,66	0,43	E
189	|10111101|	90	49	17	77	6	0	86	6	8	83	92	0,00	2,68	0,21	0,50	E
197	|11000101|	90	182	32	31	37	5	30	38	27	63	81	0,22	0,93	1,32	1,65	m
199	|11000111|	90	127	31	50	19	1	35	20	43	69	75	0,03	1,11	0,70	2,66	M
201	|11001001|	90	331	23	13	64	2	55	32	11	82	83	0,08	1,72	1,11	0,67	e
203	|11001011|	90	159	36	35	30	9	44	31	16	65	78	0,39	1,38	1,08	0,97	e
205	|11001101|	90	127	20	31	49	0	35	55	9	74	85	0,00	1,11	1,89	0,58	h
207	|11001111|	90	58	16	67	17	0	36	31	33	74	85	0,00	1,13	1,07	2,01	m
209	|11010001|	90	256	29	32	38	3	38	41	18	65	82	0,14	1,18	1,42	1,08	h
211	|11010011|	90	146	27	31	42	5	28	40	27	70	87	0,21	0,88	1,39	1,64	m
213	|11010101|	90	102	24	53	23	3	62	25	11	80	77	0,13	1,93	0,84	0,66	E
215	|11010111|	90	32	20	50	31	0	47	34	19	87	83	0,00	1,46	1,18	1,15	e
217	|11011001|	90	224	15	13	72	3	15	75	7	69	92	0,12	0,47	2,57	0,44	H
219	|11011011|	90	75	19	40	42	0	33	44	23	75	91	0,00	1,04	1,51	1,39	h
221	|11011101|	90	55	14	43	43	5	44	42	9	88	96	0,24	1,36	1,44	0,56	h
227	|11100011|	90	141	29	44	27	3	39	21	37	71	79	0,13	1,22	0,73	2,26	M
229	|11100101|	90	109	27	52	21	0	46	23	31	76	77	0,00	1,43	0,79	1,92	m
231	|11100111|	90	53	23	51	26	0	42	32	26	85	68	0,00	1,30	1,10	1,62	m
233	|11101001|	70	118	25	52	24	0	63	21	16	69	89	0,00	2,07	0,70	0,98	E
235	|11101011|	90	44	21	67	11	0	75	11	14	84	85	0,00	2,34	0,39	0,84	E
237	|11101101|	90	54	24	53	23	2	50	22	26	78	92	0,08	1,56	0,76	1,59	m
241	|11110001|	90	139	31	52	16	1	54	12	33	66	89	0,06	1,69	0,40	2,03	m
243	|11110011|	90	70	23	54	23	1	61	21	16	74	91	0,06	1,92	0,74	0,97	E
245	|11110101|	90	50	22	65	13	4	76	8	12	80	100	0,18	2,38	0,27	0,74	E
249	|11111001|	90	48	21	59	20	0	69	21	10	77	88	0,00	2,15	0,71	0,64	E
273	|100010001|	90	245	31	10	59	10	11	64	15	45	84	0,43	0,36	2,19	0,93	H
275	|100010011|	90	237	22	9	69	6	8	70	15	51	90	0,28	0,26	2,42	0,91	H
277	|100010101|	90	159	31	33	36	4	40	38	18	66	82	0,17	1,24	1,32	1,12	h
279	|100010111|	90	98	27	37	36	4	38	34	24	67	91	0,18	1,18	1,16	1,50	m
281	|100011001|	90	157	27	13	60	5	20	61	14	48	88	0,23	0,62	2,10	0,86	H
283	|100011011|	90	133	20	27	53	1	32	52	15	67	89	0,03	1,01	1,78	0,92	h
285	|100011101|	90	50	44	43	13	0	56	12	32	51	76	0,00	1,75	0,41	1,97	m
291	|100100011|	90	151	25	17	58	5	15	54	25	62	89	0,23	0,48	1,86	1,55	h
293	|100100101|	90	135	35	26	40	7	25	36	33	55	80	0,30	0,79	1,22	2,00	m
295	|100100111|	90	113	19	43	38	0	44	38	18	78	91	0,00	1,38	1,31	1,09	e

HYDROPHOBIC CLUSTER P- code	HYDROPHOBIC CLUSTER binary code	redondancy	number	frequency APC rule C	frequency APC rule E	frequency APC rule H	frequency OPS rule C	frequency OPS rule E	frequency OPS rule H	frequency OPS rule M	coverage (OPS rule) E	coverage (OPS rule) H	propensities (OPS rule) C	propensities (OPS rule) E	propensities (OPS rule) H	propensities (OPS rule) M	affinity
**297**	|100101001|	90	125	39	30	31	8	31	28	33	60	73	0,35	0,98	0,96	2,01	**M**
**299**	|100101011|	90	95	27	48	25	3	45	18	34	77	77	0,14	1,41	0,61	2,07	**m**
**301**	|100101101|	90	65	28	57	14	2	65	15	18	73	71	0,07	2,02	0,53	1,13	**e**
**303**	|100101111|	90	35	22	55	24	0	49	14	37	75	100	0,00	1,52	0,49	2,28	**m**
**305**	|100110001|	90	228	21	50	30	5	15	63	16	68	88	0,23	0,48	2,17	1,00	**H**
**307**	|100110011|	70	144	13	10	77	2	13	80	5	67	93	0,09	0,43	2,64	0,30	**H**
**309**	|100110101|	90	97	29	41	30	2	52	30	16	65	85	0,09	1,61	1,03	1,01	**e**
**311**	|100110111|	90	39	15	23	62	3	23	64	10	79	96	0,11	0,72	2,20	0,63	**H**
**313**	|100111001|	90	89	26	34	40	2	46	39	12	62	93	0,10	1,44	1,35	0,76	**e**
**315**	|100111011|	85	46	15	29	56	0	30	59	11	74	93	0,00	0,97	1,99	0,67	**H**
**325**	|101000101|	75	111	36	40	23	7	35	20	38	69	78	0,31	1,15	0,66	2,31	**M**
**327**	|101000111|	90	63	36	41	24	2	44	21	33	61	80	0,07	1,39	0,71	2,05	**m**
**329**	|101001001|	90	123	36	29	36	6	32	35	28	59	75	0,25	0,99	1,20	1,70	**m**
**331**	|101001011|	90	70	32	43	25	1	46	21	31	63	83	0,06	1,43	0,74	1,93	**m**
**333**	|101001101|	90	71	31	33	35	4	30	35	31	70	75	0,19	0,92	1,21	1,90	**m**
**335**	|101001111|	90	34	30	61	9	0	59	9	32	72	63	0,00	1,84	0,30	1,99	**m**
**337**	|101010001|	85	153	33	39	28	3	35	27	34	60	84	0,14	1,13	0,93	2,08	**m**
**339**	|101010011|	90	60	22	32	46	0	35	38	27	71	94	0,00	1,09	1,32	1,64	**m**
**341**	|101010101|	90	48	22	57	21	4	50	19	27	88	83	0,18	1,56	0,64	1,66	**m**
**345**	|101011001|	90	80	25	37	37	0	45	38	18	65	86	0,00	1,41	1,29	1,07	**e**
**355**	|101100011|	90	83	37	34	29	6	41	25	28	55	78	0,27	1,28	0,87	1,70	**m**
**357**	|101100101|	90	83	28	35	37	4	31	41	24	81	76	0,16	0,98	1,41	1,48	**m**
**359**	|101100111|	80	33	20	35	45	0	33	42	24	82	89	0,00	1,08	1,42	1,48	**m**
**361**	|101101001|	90	71	30	49	21	0	48	21	31	71	70	0,00	1,50	0,73	1,90	**m**
**363**	|101101011|	80	32	20	61	18	0	59	13	28	80	92	0,00	1,92	0,42	1,72	**e**
**369**	|101110001|	90	62	26	45	29	2	52	31	16	70	80	0,07	1,61	1,05	0,99	**e**
**371**	|101110011|	90	57	20	23	57	2	37	54	7	62	95	0,08	1,15	1,87	0,43	**h**
**393**	|110001001|	90	182	30	15	55	4	15	54	26	51	82	0,19	0,46	1,87	1,62	**h**
**395**	|110001011|	90	111	26	26	48	6	19	44	31	71	87	0,28	0,59	1,51	1,88	**m**
**397**	|110001101|	90	81	26	32	42	4	36	38	22	69	88	0,16	1,12	1,31	1,36	**m**
**399**	|110001111|	90	42	29	56	16	5	52	12	31	72	84	0,21	1,64	0,41	1,90	**m**
**401**	|110010001|	90	230	22	9	69	4	12	67	16	46	91	0,19	0,38	2,31	0,99	**H**
**403**	|110010011|	90	155	18	11	71	3	10	69	18	61	90	0,11	0,32	2,37	1,11	**H**
**405**	|110010101|	90	75	21	33	46	4	31	48	17	79	88	0,18	0,96	1,65	1,06	**h**
**407**	|110010111|	90	39	14	23	64	3	23	59	15	81	94	0,11	0,72	2,02	0,94	**H**
**409**	|110011001|	90	164	13	11	76	2	11	76	12	75	91	0,08	0,34	2,59	0,71	**H**
**411**	|110011011|	90	69	8	7	84	0	9	84	7	70	95	0,00	0,27	2,88	0,45	**H**
**413**	|110011101|	85	38	22	26	52	0	29	53	18	54	95	0,00	0,92	1,78	1,13	**h**
**419**	|110100011|	90	93	24	27	49	3	27	52	18	71	87	0,14	0,84	1,77	1,12	**h**
**421**	|110100101|	90	86	37	40	23	5	35	17	43	62	64	0,21	1,09	0,60	2,64	**M**
**423**	|110100111|	90	50	17	40	43	2	34	38	26	80	91	0,09	1,06	1,30	1,60	**m**
**425**	|110101001|	90	87	27	48	26	1	49	29	21	73	78	0,05	1,54	0,99	1,27	**e**
**427**	|110101011|	90	37	23	51	26	3	49	24	24	80	89	0,12	1,52	0,83	1,49	**e**
**433**	|110110001|	90	101	16	17	67	1	16	67	16	63	93	0,04	0,50	2,31	0,97	**H**
**435**	|110110011|	75	72	7	26	67	0	25	68	7	94	94	0,00	0,82	2,26	0,42	**H**
**437**	|110110101|	90	30	27	35	38	0	47	40	13	65	84	0,00	1,46	1,37	0,82	**e**
**441**	|110111001|	90	33	8	20	72	0	18	76	6	81	96	0,00	0,57	2,60	0,37	**H**
**453**	|111000101|	90	68	35	42	23	0	41	28	31	62	66	0,00	1,29	0,96	1,90	**m**
**455**	|111000111|	90	39	24	53	23	3	21	15	62	67	98	0,11	0,64	0,53	3,78	**M**
**457**	|111001001|	90	86	25	32	42	3	33	42	22	73	85	0,15	1,02	1,44	1,36	**h**
**459**	|111001011|	90	45	16	30	54	0	27	47	27	76	94	0,00	0,83	1,60	1,64	**m**
**461**	|111001101|	90	33	19	43	37	0	48	27	24	73	90	0,00	1,52	0,94	1,49	**e**
**465**	|111010001|	90	60	30	44	26	5	40	22	33	70	79	0,22	1,25	0,74	2,05	**m**
**467**	|111010011|	90	39	21	32	47	0	36	46	18	72	85	0,00	1,12	1,58	1,10	**h**
**473**	|111011001|	90	58	12	37	50	0	40	52	9	84	92	0,00	1,24	1,78	0,53	**h**
**483**	|111100011|	90	45	30	45	25	2	40	20	38	65	79	0,10	1,25	0,69	2,32	**M**
**485**	|111100101|	90	34	18	70	12	0	68	9	24	82	93	0,00	2,11	0,30	1,44	**e**
**489**	|111101001|	75	31	22	59	19	0	55	0	45	71	0	0,00	1,80	0,00	2,76	**m**
**497**	|111110001|	90	30	27	60	13	0	53	10	37	67	85	0,00	1,67	0,34	2,25	**m**

HYDROPHOBIC CLUSTER		redondancy	number	frequency									propensities (OPS rule)				affinity
				APC rule			OPS rule				coverage (OPS rule)						
P- code	binary code			C	E	H	C	E	H	M	E	H	C	E	H	M	
547	|1000100011|	90	82	35	18	46	5	18	44	33	48	78	0,22	0,57	1,51	2,02	m
549	|1000100101|	90	108	34	32	34	6	31	37	27	70	77	0,25	0,95	1,27	1,65	m
551	|1000100111|	90	42	29	33	38	7	29	36	29	73	87	0,32	0,89	1,23	1,75	m
553	|1000101001|	90	93	40	32	28	4	30	25	41	62	59	0,19	0,94	0,85	2,51	M
555	|1000101011|	90	46	29	37	34	0	39	26	35	69	68	0,00	1,22	0,90	2,14	m
557	|1000101101|	90	47	34	38	28	2	38	28	32	57	82	0,09	1,20	0,95	1,96	m
561	|1000110001|	90	99	37	20	43	4	22	45	28	52	77	0,18	0,69	1,56	1,74	m
563	|1000110011|	90	64	19	10	70	0	9	77	14	53	87	0,00	0,29	2,63	0,86	H
565	|1000110101|	90	60	36	47	17	0	60	18	22	60	77	0,00	1,88	0,63	1,33	e
569	|1000111001|	90	59	27	31	42	0	41	37	22	59	85	0,00	1,27	1,28	1,35	m
573	|1000111101|	90	48	33	59	9	0	63	8	29	63	90	0,00	1,95	0,29	1,79	e
581	|1001000101|	90	108	32	14	54	8	11	60	20	56	80	0,37	0,35	2,07	1,25	h
583	|1001000111|	90	44	33	32	35	2	25	32	41	58	80	0,10	0,78	1,09	2,51	M
585	|1001001001|	90	95	35	17	48	3	16	43	38	52	75	0,14	0,49	1,48	2,33	m
587	|1001001011|	90	36	40	27	33	6	25	33	36	52	65	0,25	0,78	1,14	2,22	M
589	|1001001101|	90	69	25	18	56	3	20	57	20	61	84	0,13	0,63	1,94	1,25	h
593	|1001010001|	90	83	44	25	31	13	19	30	37	54	72	0,59	0,60	1,03	2,29	M
595	|1001010011|	90	47	31	28	41	0	34	40	26	61	73	0,00	1,06	1,39	1,57	m
597	|1001010101|	90	33	30	42	27	3	52	27	18	67	83	0,13	1,61	0,94	1,12	e
599	|1001010111|	90	31	29	42	29	0	48	16	35	64	82	0,00	1,51	0,55	2,18	m
601	|1001011001|	90	44	23	24	53	0	27	64	9	76	80	0,00	0,85	2,18	0,56	H
611	|1001100011|	90	54	31	20	49	7	22	39	31	63	85	0,33	0,69	1,33	1,93	m
613	|1001100101|	90	92	25	18	57	1	13	54	32	74	80	0,05	0,41	1,87	1,94	m
617	|1001101001|	90	58	35	26	39	2	24	38	36	52	74	0,08	0,75	1,30	2,22	m
621	|1001101101|	90	31	23	31	46	6	29	45	19	83	85	0,29	0,91	1,55	1,19	h
625	|1001110001|	90	45	30	33	36	0	38	40	22	65	73	0,00	1,18	1,37	1,36	h
649	|1010001001|	90	92	37	25	39	7	20	34	40	57	74	0,29	0,61	1,16	2,47	M
651	|1010001011|	90	43	42	34	23	5	26	21	49	57	62	0,21	0,80	0,72	3,00	M
653	|1010001101|	90	43	37	34	29	0	33	26	42	44	85	0,00	1,02	0,88	2,57	M
657	|1010010001|	90	79	28	23	48	6	22	47	25	72	82	0,28	0,67	1,61	1,55	h
659	|1010010011|	85	53	28	12	60	4	9	53	34	54	76	0,17	0,30	1,79	2,08	m
661	|1010010101|	90	52	35	51	14	8	48	12	33	70	77	0,34	1,50	0,40	2,01	m
665	|1010011001|	90	78	27	15	58	5	15	56	23	63	82	0,23	0,48	1,94	1,42	h
667	|1010011011|	90	33	21	12	67	3	12	64	21	60	83	0,13	0,38	2,18	1,30	h
675	|1010100011|	90	53	35	30	34	2	17	32	49	61	76	0,08	0,53	1,10	3,01	M
677	|1010100101|	90	49	32	47	21	2	41	24	33	72	73	0,09	1,28	0,84	2,01	m
681	|1010101001|	90	55	29	44	27	5	45	22	27	69	91	0,24	1,42	0,75	1,67	m
689	|1010110001|	90	63	32	45	23	3	46	22	29	68	86	0,14	1,44	0,76	1,75	m
709	|1011000101|	90	53	33	40	27	0	34	30	36	69	71	0,00	1,06	1,04	2,20	M
713	|1011001001|	90	62	25	24	51	0	13	55	32	54	83	0,00	0,40	1,88	1,98	m
721	|1011010001|	80	33	34	36	29	3	33	24	39	63	74	0,13	1,08	0,81	2,41	M
729	|1011011001|	90	35	28	34	38	3	49	26	23	59	91	0,13	1,52	0,88	1,40	e
785	|1100010001|	90	81	30	10	60	4	9	58	30	61	81	0,16	0,27	1,99	1,82	h
787	|1100010011|	90	67	18	9	74	3	6	73	18	50	90	0,13	0,19	2,51	1,10	H
789	|1100010101|	90	44	24	37	39	0	27	34	39	74	89	0,00	0,85	1,17	2,37	M
793	|1100011001|	90	59	26	17	57	3	17	56	24	65	83	0,15	0,53	1,92	1,46	h
795	|1100011011|	90	31	21	21	58	0	13	58	29	53	88	0,00	0,40	1,99	1,78	h
803	|1100100011|	90	41	24	11	65	0	15	61	24	38	86	0,00	0,46	2,09	1,50	h
805	|1100100101|	90	71	30	23	47	6	20	48	27	74	80	0,25	0,62	1,64	1,64	H
807	|1100100111|	90	33	22	38	40	3	33	36	27	78	89	0,13	1,04	1,25	1,67	m
809	|1100101001|	90	52	32	37	31	4	35	21	40	68	89	0,17	1,08	0,73	2,48	M
811	|1100101011|	90	40	21	42	37	0	33	20	48	75	91	0,00	1,02	0,69	2,92	M
817	|1100110001|	90	64	24	8	68	5	11	73	11	67	82	0,21	0,34	2,52	0,67	H
819	|1100110011|	90	34	9	5	86	0	0	88	12	0	92	0,00	0,00	3,03	0,72	H
821	|1100110101|	90	31	24	28	48	0	26	52	23	84	81	0,00	0,81	1,77	1,39	h
837	|1101000101|	90	53	28	36	36	0	23	32	45	78	76	0,00	0,71	1,10	2,78	M
841	|1101001001|	90	62	39	30	31	3	18	23	56	60	64	0,14	0,55	0,77	3,47	M
849	|1101010001|	80	42	26	50	24	0	43	31	26	82	66	0,00	1,39	1,04	1,60	m
905	|1110001001|	90	52	42	31	27	6	42	29	23	52	79	0,26	1,32	0,99	1,42	m

HYDROPHOBIC CLUSTER		redondancy	number	frequency													affinity
				APC rule			OPS rule				coverage (OPS rule)		propensities (OPS rule)				
P- code	binary code			C	E	H	C	E	H	M	E	H	C	E	H	M	
913	|1110010001|	90	53	29	25	46	4	23	49	25	63	81	0,17	0,71	1,68	1,51	h
915	|1110010011|	85	39	14	31	54	3	15	41	41	98	96	0,11	0,49	1,39	2,51	M
921	|1110011001|	90	37	24	23	53	3	22	59	16	58	87	0,12	0,68	2,04	1,00	H
965	|1111000101|	90	39	29	50	21	0	38	21	41	70	68	0,00	1,20	0,70	2,52	M
1093	|10001000101|	75	56	35	22	43	5	11	48	36	61	78	0,23	0,35	1,60	2,18	m
1097	|10001001001|	90	64	29	7	64	2	6	70	22	34	81	0,07	0,20	2,41	1,34	H
1101	|10001001101|	90	53	24	15	61	2	6	57	36	70	85	0,08	0,18	1,94	2,20	m
1105	|10001010001|	90	53	28	19	53	6	13	51	30	68	83	0,25	0,41	1,75	1,85	m
1113	|10001011001|	90	48	20	12	67	0	15	69	17	55	88	0,00	0,46	2,36	1,02	H
1125	|10001100101|	90	31	41	21	37	0	16	42	42	44	67	0,00	0,50	1,44	2,58	M
1129	|10001101001|	90	32	29	32	39	3	16	22	59	65	88	0,14	0,49	0,75	3,65	M
1161	|10010001001|	90	86	20	8	72	2	6	77	15	58	86	0,10	0,18	2,63	0,93	H
1163	|10010001011|	85	50	25	18	57	0	10	50	40	40	88	0,00	0,32	1,69	2,45	m
1165	|10010001101|	90	40	24	16	60	3	10	60	28	57	85	0,11	0,31	2,06	1,69	h
1169	|10010010001|	90	79	26	14	60	3	13	61	24	52	84	0,11	0,40	2,09	1,48	h
1171	|10010010011|	90	47	28	9	62	6	6	62	26	58	78	0,28	0,20	2,12	1,57	h
1173	|10010010101|	90	38	28	42	30	0	37	21	42	68	82	0,00	1,15	0,72	2,59	M
1177	|10010011001|	90	58	17	5	78	2	3	74	21	36	88	0,08	0,11	2,54	1,27	H
1193	|10010101001|	90	42	34	34	32	0	33	31	36	60	73	0,00	1,04	1,06	2,19	M
1221	|10011000101|	90	51	25	10	65	2	10	73	16	58	80	0,09	0,31	2,49	0,96	H
1225	|10011001001|	90	58	13	2	85	0	0	86	14	0	89	0,00	0,00	2,96	0,85	H
1233	|10011010001|	90	54	27	18	56	0	20	63	17	58	82	0,00	0,64	2,16	1,02	H
1241	|10011011001|	90	34	10	3	87	0	26	52	23	84	81	0,00	0,81	1,77	1,39	h
1297	|10100010001|	90	50	35	21	44	2	18	40	40	54	79	0,09	0,56	1,37	2,46	M
1299	|10100010011|	90	43	37	18	45	7	12	44	37	49	86	0,31	0,36	1,52	2,29	m
1305	|10100011001|	90	42	26	21	54	0	12	52	36	62	84	0,00	0,37	1,80	2,19	m
1321	|10100101001|	90	40	32	43	25	5	33	13	50	73	69	0,22	1,02	0,43	3,07	M
1329	|10100110001|	90	43	21	19	60	2	14	51	33	85	83	0,10	0,44	1,76	2,00	m
1349	|10101000101|	85	39	44	37	19	3	26	18	54	44	68	0,11	0,82	0,61	3,30	M
1353	|10101001001|	85	40	38	27	35	3	33	33	33	46	83	0,11	1,04	1,10	1,99	m
1417	|10110001001|	90	57	30	23	47	7	23	32	39	53	85	0,31	0,71	1,08	2,37	M
1425	|10110010001|	90	45	23	14	63	2	11	58	29	47	90	0,10	0,35	1,98	1,77	h
1571	|11000100011|	80	38	35	23	41	0	11	32	58	50	73	0,00	0,34	1,06	3,54	M
1573	|11000100101|	90	32	37	28	35	0	25	38	38	51	70	0,00	0,78	1,29	2,30	M
1577	|11000101001|	90	33	37	33	30	3	15	18	64	49	86	0,13	0,47	0,62	3,91	M
1585	|11000110001|	90	33	29	31	40	0	24	36	39	80	73	0,00	0,76	1,25	2,42	M
1605	|11001000101|	90	51	32	22	46	4	10	47	39	75	75	0,17	0,31	1,61	2,41	m
1609	|11001001001|	90	31	30	11	59	0	3	52	45	55	75	0,00	0,10	1,77	2,77	m
1617	|11001010001|	90	30	29	33	38	0	30	27	43	58	90	0,00	0,94	0,92	2,66	M
1637	|11001100101|	90	33	30	9	61	12	15	67	6	60	84	0,54	0,47	2,29	0,37	H
1673	|11010001001|	90	72	29	17	55	3	10	54	33	58	85	0,12	0,30	1,86	2,05	m
1809	|11100010001|	90	32	24	28	48	3	9	44	44	45	64	0,14	0,29	1,50	2,69	M
2185	|100010001001|	90	47	28	5	67	9	4	66	21	42	82	0,38	0,13	2,26	1,31	h
2193	|100010010001|	90	71	17	7	77	3	1	75	21	67	92	0,12	0,04	2,56	1,30	H
2195	|100010010011|	90	34	12	4	85	0	0	82	18	0	93	0,00	0,00	2,83	1,08	H
2197	|100010010101|	90	30	30	44	26	0	23	13	63	60	77	0,00	0,73	0,46	3,89	M
2201	|100010011001|	90	54	10	6	84	0	6	87	7	64	93	0,00	0,17	2,99	0,45	H
2225	|100010110001|	90	32	19	16	65	6	6	59	28	46	97	0,28	0,20	2,04	1,73	h
2265	|100011011001|	90	34	17	14	68	0	9	68	24	61	91	0,00	0,28	2,32	1,44	h
2321	|100100010001|	90	49	24	7	69	2	0	63	35	0	88	0,09	0,00	2,17	2,13	h
2323	|100100010011|	90	37	11	7	82	3	0	70	27	0	97	0,12	0,00	2,41	1,66	h
2353	|100100110001|	90	46	24	13	63	2	15	65	17	58	85	0,10	0,48	2,24	1,07	H
2449	|100110010001|	90	79	15	3	82	4	1	86	9	100	89	0,17	0,04	2,95	0,54	H
2705	|101010010001|	90	38	36	25	39	8	13	37	42	48	70	0,35	0,41	1,26	2,59	M
3141	|110001000101|	70	29	36	34	30	0	10	21	69	61	75	0,00	0,34	0,69	4,21	M
3209	|110010001001|	90	46	19	7	74	4	11	70	15	42	92	0,19	0,34	2,39	0,93	H
4369	|1000100010001|	90	30	25	4	71	3	3	77	17	54	83	0,15	0,10	2,63	1,02	H
4371	|1000100010011|	90	34	15	4	81	3	3	68	26	23	93	0,13	0,09	2,32	1,63	h
