# Supplementary material for: A generalized analysis of hydrophobic and loop clusters within globular protein sequences
Source: BMC Struct Biol. 2007 Jan 8;7:2. doi: 10.1186/1472-6807-7-2 (PMC1774571; doi:10.1186/1472-6807-7-2)
Supplement: Additional File 5 — Loop clusters analysis. Structural features of 167 loop cluster species, which are frequently encountered in protein globular domains. The considered redundancy levels and the occurrences within each loop cluster species are indicated. Frequencies of association with secondary structures (CONSENSUS assignment) are given according to the APC rule (see text). # indicates a position for which no coordinate has been reported in the analyzed PDB files. This table can also be found on our Web server [24]. [file 1472-6807-7-2-S5.pdf]

LOOP CLUSTER		redundancy	cluster number	APC score CONSENSUS			
P-code	binary code			c	E	H	#
1	|1|	90	34270	52	13	30	5
3	|11|	90	16901	65	9	21	4
5	|101|	90	9521	63	11	24	3
7	|111|	90	7113	67	10	17	6
9	|1001|	90	4657	56	12	27	4
11	|1011|	90	3667	65	12	19	4
13	|1101|	90	3705	66	12	18	3
15	|1111|	75	2346	70	11	15	4
17	|10001|	90	1967	52	13	31	5
19	|10011|	90	1387	64	12	20	4
21	|10101|	90	1569	65	12	20	3
23	|10111|	90	1029	69	12	14	5
25	|11001|	90	1491	62	12	22	4
27	|11011|	85	1028	70	10	15	5
29	|11101|	90	1054	68	12	16	4
31	|11111|	90	777	73	12	10	5
35	|100011|	70	633	57	16	23	4
37	|100101|	90	737	61	12	22	5
39	|100111|	75	434	64	11	20	5
41	|101001|	90	762	59	12	25	4
43	|101011|	90	461	67	12	17	4
45	|101101|	90	470	66	12	18	4
47	|101111|	90	318	72	12	11	4
49	|110001|	90	709	54	13	28	5
51	|110011|	90	466	68	11	16	4
53	|110101|	90	440	66	11	20	3
55	|110111|	90	347	72	12	12	4
57	|111001|	90	510	63	10	21	6
59	|111011|	90	303	69	10	14	7
61	|111101|	90	358	65	12	18	5
63	|111111|	75	213	71	14	11	4
69	|1000101|	85	334	53	16	26	5
71	|1000111|	90	228	60	15	20	5
73	|1001001|	90	350	52	12	29	6
75	|1001011|	90	214	63	14	18	5
77	|1001101|	90	210	64	17	16	3
79	|1001111|	90	151	62	15	14	8
81	|1010001|	90	289	56	14	25	5
83	|1010011|	90	269	68	14	17	2
85	|1010101|	90	205	63	14	18	5
87	|1010111|	90	153	71	11	14	4
89	|1011001|	90	257	60	14	20	6
91	|1011011|	75	128	68	12	15	4
93	|1011101|	90	147	65	16	16	3
95	|1011111|	90	73	70	17	10	2
99	|1100011|	90	280	64	10	22	4
101	|1100101|	90	218	60	12	23	5
103	|1100111|	90	148	66	10	18	7
105	|1101001|	90	282	59	13	24	5
107	|1101011|	90	149	72	8	16	4
109	|1101101|	90	146	69	12	14	5
111	|1101111|	90	99	70	13	13	5
113	|1110001|	90	253	54	15	25	6
115	|1110011|	75	157	67	12	15	6
117	|1110101|	90	160	69	10	17	4
119	|1110111|	90	84	72	9	15	4

LOOP CLUSTER		redundancy	cluster number	APC score CONSENSUS			
P-code	binary code			c	E	H	#
121	|1111001|	90	184	62	19	17	2
123	|1111011|	75	102	67	13	16	4
125	|1111101|	90	102	68	14	11	7
127	|1111111|	70	50	63	12	7	19
137	|10001001|	90	159	52	14	29	5
139	|10001011|	90	102	62	14	19	5
141	|10001101|	90	107	55	18	18	8
143	|10001111|	90	76	58	16	22	4
145	|10010001|	90	155	50	15	29	6
147	|10010011|	90	121	58	13	23	6
149	|10010101|	90	110	66	10	20	4
151	|10010111|	90	78	65	15	8	12
153	|10011001|	90	86	59	10	21	11
155	|10011011|	90	54	72	14	13	1
157	|10011101|	90	58	56	24	16	3
163	|10100011|	90	116	63	16	16	5
165	|10100101|	90	119	62	11	20	7
167	|10100111|	90	79	65	15	15	4
169	|10101001|	90	120	60	10	23	7
171	|10101011|	90	80	68	11	16	5
173	|10101101|	90	71	67	11	20	2
175	|10101111|	80	40	65	14	14	7
177	|10110001|	90	103	57	17	20	7
179	|10110011|	90	83	64	16	15	5
181	|10110101|	90	79	75	8	12	5
183	|10110111|	90	48	67	14	10	9
185	|10111001|	90	64	56	10	18	15
187	|10111011|	90	58	64	21	11	4
189	|10111101|	90	54	76	14	9	1
197	|11000101|	90	140	59	13	24	4
199	|11000111|	90	94	63	13	16	8
201	|11001001|	90	120	55	13	26	6
203	|11001011|	90	86	65	11	17	7
205	|11001101|	80	93	69	9	16	7
207	|11001111|	90	60	74	10	9	6
209	|11010001|	90	150	55	15	26	4
211	|11010011|	90	87	61	13	18	8
213	|11010101|	90	88	66	10	18	6
215	|11010111|	90	41	73	8	17	2
217	|11011001|	90	57	60	10	23	7
219	|11011011|	90	33	79	8	9	5
221	|11011101|	90	44	68	13	14	4
227	|11100011|	75	88	59	15	23	4
229	|11100101|	90	82	66	13	17	4
231	|11100111|	90	47	65	9	20	6
233	|11101001|	90	76	64	8	22	6
235	|11101011|	90	54	71	13	12	4
237	|11101101|	90	42	77	9	9	5
239	|11101111|	90	30	68	10	17	5
241	|11110001|	90	83	61	12	19	8
243	|11110011|	90	52	62	12	23	4
245	|11110101|	90	56	65	11	15	8
249	|11111001|	90	43	67	8	16	9
251	|11111011|	90	36	75	10	12	3
253	|11111101|	85	34	65	14	13	8
273	|100010001|	90	94	47	12	33	9

LOOP CLUSTER		redundancy	cluster number	APC score CONSENSUS			
P-code	binary code			c	E	H	#
275	|100010011|	90	54	63	16	15	5
277	|100010101|	90	50	64	12	18	5
279	|100010111|	90	33	58	18	11	13
281	|100011001|	90	39	61	15	21	3
291	|100100011|	90	68	55	10	28	7
293	|100100101|	90	55	59	8	32	2
295	|100100111|	90	32	58	19	14	9
297	|100101001|	90	49	52	13	26	9
299	|100101011|	90	34	67	9	15	9
305	|100110001|	90	60	57	15	24	4
307	|100110011|	90	35	69	12	13	5
313	|100111001|	90	32	64	13	16	8
325	|101000101|	90	72	55	17	23	5
327	|101000111|	90	44	60	16	18	6
329	|101001001|	90	50	55	14	26	4
331	|101001011|	90	41	69	9	20	2
333	|101001101|	90	37	65	14	15	6
337	|101010001|	90	51	58	17	22	4
339	|101010011|	90	41	69	14	14	3
355	|101100011|	90	62	62	16	18	4
357	|101100101|	90	30	60	11	19	10
359	|101100111|	90	31	70	18	8	4
361	|101101001|	90	32	61	16	18	5
363	|101101011|	90	38	71	10	12	7
365	|101101101|	90	32	72	11	12	6
369	|101110001|	90	35	60	18	16	6
393	|110001001|	90	54	46	12	34	8
395	|110001011|	90	49	50	11	24	14
397	|110001101|	90	46	64	15	14	7
399	|110001111|	90	38	64	17	16	3
401	|110010001|	90	65	55	12	29	4
403	|110010011|	90	44	57	20	21	2
405	|110010101|	90	37	64	12	11	13
419	|110100011|	90	48	59	16	21	4
421	|110100101|	90	38	62	19	19	1
423	|110100111|	90	30	64	11	11	13
425	|110101001|	90	53	56	14	25	5
433	|110110001|	90	49	55	15	18	13
437	|110110101|	90	33	69	11	15	4
453	|111000101|	90	57	61	14	22	4
455	|111000111|	90	44	59	13	17	11
457	|111001001|	90	35	62	11	26	2
459	|111001011|	90	51	27	5	6	62
461	|111001101|	90	31	65	10	20	5
465	|111010001|	90	35	63	9	25	2
483	|111100011|	85	41	60	17	14	9
485	|111100101|	90	38	62	16	16	6
547	|1000100011|	90	38	54	16	24	7
553	|1000101001|	90	38	56	13	25	6
581	|1001000101|	90	35	53	11	26	10
585	|1001001001|	90	35	54	15	19	11
613	|1001100101|	90	32	59	16	17	8
649	|1010001001|	90	44	53	12	20	15
657	|1010010001|	90	38	54	16	27	2
659	|1010010011|	90	30	61	12	25	2
