# Supplementary material for: A generalized analysis of hydrophobic and loop clusters within globular protein sequences
Source: BMC Struct Biol. 2007 Jan 8;7:2. doi: 10.1186/1472-6807-7-2 (PMC1774571; doi:10.1186/1472-6807-7-2)

A) Pdb 1dc1_A

```
Sequence   PKDLLYLEDIQDALLYASGISD
Consensus  HHHHHHcHHHHHHHHHHHcccc
PSI-PRED   HHHHHHHHHHHHHHHHHccccc
```

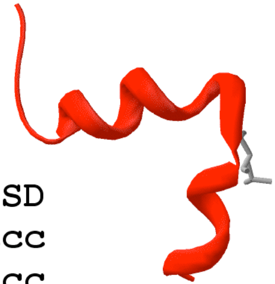

B) Pdb 1b43_A

```
Sequence   KPELIILEEVLKELKLTREK
Consensus  ccEEEEHHHHHHHHcccHHH
PSI-PRED   ccHHHHHHHHHHHHHHHHHH
```

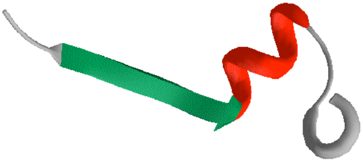

C) Pdb 1dkx_A

```
Sequence   TPLSLGIETMGGVMTTLIAK
Consensus  cccEEEEEEcccEEEEEEcc
PSI-PRED   cHHHHHHHHHHHHHHHHHHc
```

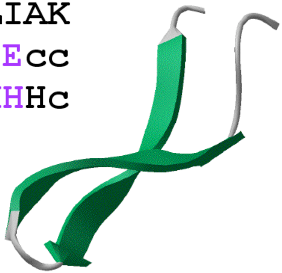

Supplement: Additional File 7 — Three examples of PSI-PRED overprediction of helices, at the detriment of the multiple assignment (Consensus line). [file 1472-6807-7-2-S7.pdf]
